# How do firms value sales career paths?

**DOI:** 10.1007/s11747-023-00952-4

**Published:** 2023-06-17

**Authors:** Ali Reza Keshavarz, Dominique Rouzies, Francis Kramarz, Bertrand Quelin, Michael Segalla

**Affiliations:** 1 School of Business, Maynooth University, Co. Kildare, Ireland; 2 HEC Paris, 1 Avenue de La Liberation, 78351 Jouy-en-Josas, France; 3 BMI Executive Institute, Konstitucijos Ave 7, Vilnius, Lithuania; 4 BMI Executive Institute, Avenue Louise 65, 1050 Ixelles, Brussels, Belgium; 5 CREST-ENSAE, 5 Avenue Henry Le Chatelier, 91120 Palaiseau, France; 6 Uppsala University, Kyrkogårdsgatan 10, 753 13 Uppsala, Sweden

**Keywords:** Salesforce compensation, Career paths, Experience, Job signal, Skills

## Abstract

Sales employees can build their careers across industries, firms, and occupations. Yet it is unclear how their sales career paths affect their compensation. To assess the value of their experience, we examine the paychecks drawn by nearly 25,000 sales employees over 22 years. Consistent with our arguments, we find that firms place greater value on sales managers' experience than salespeople’s, reflecting the multiplier effect attributed to managers. In particular, *sales occupation experience* seems to be the most salient type of experience for both groups, as it is fungible across industries and firms. We uncover two distinct paths in sales organizations: the salesperson’s career path rewarding sales experience and another path providing promotional prospects in sales. Analyses indicate that most newly promoted sales managers have no sales experience and move laterally from other managerial positions. Implications of our findings for research and sales-compensation practice are discussed.

**Supplementary Information:**

The online version contains supplementary material available at 10.1007/s11747-023-00952-4.

A recent survey indicates that employees switch roles every two to four years on average (i.e., McKinsey Global Institute, 2022). Sales jobs have long been characterized by one of the highest rates of turnover among occupations (Richardson, 1999), and many salespeople constantly keep a lookout for new jobs with better pay and professional development prospects (Charles & Kelly, 2017). One of the next steps in the career path of salespeople might be sales management. But are salespeople and sales managers equally confronted by the same career development decisions (i.e., whether to remain in the same industry, remain in the same firm, or even stay in the same occupation)? Perhaps most importantly, how do these career choices affect their promotion and earnings prospects?

Accurately assessing competence and setting fair compensation are crucial for employers. Discernment is especially needed when firms hire new employees whose performance track records may be unverifiable or obscured (Bidwell, 2011). Without reliable performance indicators for sales-job applicants, employers typically rely on observable characteristics such as previous work experience. For example, a company like Hertz, which posts one of the highest numbers of sales jobs at all salary levels in the USA, often requires a minimum period of *sales experience* and general *retail industry experience* (Hertz, 2021). Similarly, other companies expanding their product or solution ranges often want their salesforce to have *specific industry specializations* (Kovac, 2016).

The critical question for researchers and managers is: How do employers value the several types of employee experience at multi-tier organizations? The aim of this research is to determine the differential impact of accumulated career experiences (i.e., within a *firm*, *industry,* or *sales occupation*) on the compensation levels of sales managers and salespeople. This conceptualization is consistent with the observation that employees are sorted into skill categories that reflect the dominant notion of value used by labor-market participants (Zuckerman et al., 2003). We believe our exceptional panel of more than 5,000 sales managers and 19,000 salespeople goes a long way toward demonstrating the field validity of our insights. Our instrumental-variable analysis demonstrates that firms value sales personnel’s *sales occupation experience* much more than their *firm* or *industry experience.* Notably, we confirm the relative importance of sales managers (given their impact on sales teams) with data showing that returns to their experience, especially occupation experience, are higher than returns for salespeople. Conversely, firms apparently value firm experience when hiring sales managers and often seek to source them internally. Indeed, our findings reveal that sales managers and salespeople do not generally share the same sales, firm, or industry experience. In fact, a job as a salesperson does not appear to be the natural first step of a sales manager. Our supplementary analysis shows that most newly promoted sales managers do not have sales experience and are moved laterally from different managerial positions.

The contributions of this paper are four-fold. First, our study revisits the issue of salesforce compensation. Despite important prior results shedding light on the selection role of salesforce compensation (Boles et al., 2012; Daljord et al., 2016; Lo et al., 2011; Zoltners et al., 2008), little attention has been paid to the mobility of salespeople and resulting compensation levels. This lack of interest is surprising, given that mobility among salespeople often exceeds that of other professions (Richardson, 1999). We draw primarily on literature from salesforce management, labor economics, human resource management, and management and industrial/organizational psychology to probe and explain the value of experience in a sales career. Using detailed information on employee occupation, firm, and job changes, we investigate the compensation level that firms establish for the specialized work experience of sales managers and salespeople. Thus, we disentangle the returns on the levels of industry, firm, and sales occupation as well as general work experience in a model of individual-level compensation.

Second, with our focus on explaining sales employees’ returns to experience, we account for the fact that mobility decisions are typically endogenous. The sample of employees who stay in a job is not random because employees actively decide if and when to change jobs. For example, if employees find a firm that fits their skills and motivations, they are likely to stay longer in the firm and command greater compensation as their performance improves. Hence, returns to firm experience will be overestimated. We use an instrumental-variable method to control for individual heterogeneity and individuals’ endogenous choices of firms, industries, and occupations (Altonji & Shakotko, 1987; Sullivan, 2010). This instrumental-variable method simultaneously controls for individual heterogeneity and the match effect of individuals' abilities and motivations to those firms, industries, and occupations. This is important because these effects (1) have largely been ignored by research specific to *sales* careers (e.g., Cron et al., 1988), and (2) are not completely controlled for by most empirical career research (e.g., Bidwell, 2011). For the rare exceptions, see Custódio et al. (2013), Leung (2014), or Zuckerman et al. (2003).

Third, we investigate whether the impact of career path on compensation at the sales-management level replicates the impact found at the salespeople level. Benson et al. (2019) suggested that when promoting salespeople to sales managers, reliance on observable characteristics of salespeople rather than their current job performance may lead to better results. However, academic accounts of the value of past experiences of sales managers are not conclusive. Of the fifteen skills, types of knowledge, and abilities necessary for sales managers outlined by Powers et al. (2014), only three appear firm-specific, and only one is industry-specific. Other scholars point to the importance of similar non-firm or non-industry-specific skills such as time management, leadership, and the ability to be a morale promoter and role model (Deeter-Schmelz et al., 2002). In contrast, more recent studies emphasize the importance of sales managers' industry and firm experiences in developing skills and abilities to leverage managerial social capital (Wang et al., 2017). Our findings contribute to this stream of research by exploring whether sales management is a continuation of a salesperson job (Anderson et al., 1999) and how different career paths are valued within a firm’s sales-management function.

Fourth, the unique characteristics of our data (sales employee careers over 22 years across firms, industries, and occupations) permit the investigation of our salesforce compensation theory that features a temporal perspective novel to marketing research (capturing nonlinear relationships between pay and accumulated experiences across industries, firms, and occupations). Understanding the impact of career history on compensation in the sales context is worthwhile, especially because about one in ten U.S. employees work in sales (U.S. Bureau of Labor Statistics 2019). We thus establish the importance of career paths for designing compensation plans.

## Compensation, seniority, experience, and career

The most relevant strands of literature for understanding the relationship between sales employee experience and compensation levels report the research on (A) salesforce compensation, (B) returns to seniority, (C) work experience, and (D) career.^1^ As shown in Table 1, each research stream has notable gaps that our research aims to fill.

**Table 1:** Research gaps at the intersection of research on careers, work experience and salesforce compensation

Research Characteristics	A-Determinants of Salesforce Compensation Level Research	B-Returns to Seniority Research	C-Work Experience Research	D-Career Research	Our Research
Human Resource Category	Salespeople	Employees, workers, executives, CEO	Employees, workers, executives, CEO	Employees, workers, executives, CEO	Salespeople and sales managers
Research Gap	Sales management jobs are generally absent from this research	Salespeople and sales management jobs are generally absent from this research	Salespeople and sales management jobs are generally absent from this research	Salespeople and sales management jobs are generally absent from this research (exceptions include Cron et. al. 1988 among others)	
Experience-Related Antecedent	e.g., Coughlan & Narasimhan, 1992, Misra et al., 2005	e.g., Altonji & Shakotko, 1987, Buchinsky et al., 2010, Serneels, 2008	e.g., Quinones 2004*; Quinones et al. 1995^*^, Tesluk & Jacobs, 1998	e.g., Bidwell & Briscoe, 2010	Industry-, firm-, sales occupation, rank level- and general work experience
Research Gap	Generally, only a few dimensions of experience	Generally, only a few dimensions of experience	Variety of dimensions of experience: measurement modes (i.e., time, amount, type, density or developmental impact, and timing), levels of specificity (i.e., task, job, organization, work group, organizational level)	Generally, only a few dimensions of experience	
Compensation Related Outcome	e.g., Coughlan & Narasimhan, 1992, Daljord et al., 2016, Misra et al., 2005	e.g., Altonji & Shakotko, 1987, Buchinsky et al., 2010, Medoff & Abraham, 1980, Serneels, 2008	e.g., Custodio et al. 2013	e.g., Bidwell, 2011, Bidwell & Mollick, 2015	Individual level compensation
Research Gap	Individual level compensation	Individual level compensation	The rare investigations of compensation issues feature only a few dimensions of work experience (e.g., Schultz et al., 2013; Slaughter et al., 2007)	Individual level compensation	
Methodology Controlling for Individual Heterogeneity	e.g., Daljord et al., 2016	e.g., Altonji & Shakotko, 1987	e.g., Leung, 2014	e.g., Bidwell, 2011, Bidwell & Mollick, 2015	Control for individual heterogeneity
Research Gap	Rare control for individual heterogeneity (when relevant)	Generally, control for individual heterogeneity	Rare control for individual heterogeneity	Rare control for individual heterogeneity	

For the sake of clarity, only experience-related antecedents of salesforce compensation level (A) and returns of experience research (B) are reported in this Table. Similarly, only compensation-related outcomes of work experience outcome research (C) and career outcome research (D) are reported in this Table; The shaded cells of this Table represent research gaps. *Meta-analysis

### Salesforce compensation research

Prior research on salesforce compensation in marketing (column A in Table 1) is mostly based on microeconomics (for recent reviews, see Chung et al., 2020; Rouziès & Onyemah, 2018) and pays scant attention to pay *levels*. Although compensation plan *structures* (e.g., Basu et al., 1985; Joseph & Kalwani, 1995) are discussed extensively in this literature, the topic of compensation *levels* remains under-researched. Furthermore, few facets of work-related experience were examined, with most studies focused on sales experience and firm experience (e.g., Coughlan & Narasimhan, 1992; Misra et al., 2005). In this regard, the salesforce literature suggests that experience has a positive and concave impact on compensation levels. Meanwhile, research on sales-*management* compensation is almost nonexistent; and little attention has been paid to endogeneity issues in this research stream.

### Returns to seniority research

Within the labor economics literature (column B in Table 1), Altonji and Shakotko (1987) were among the first to emphasize that unobserved heterogeneity across individuals and jobs may produce inconsistent estimates of the effect of experience on wages. Research on returns to seniority controlling for individual heterogeneity generally found concave and differential impact of firm and work experience on wages (e.g., Altonji & Shakotko, 1987). Furthermore, the literature suggests that these effects depend on the level of education or firm size, among other factors (e.g., Buchinsky et al., 2010; Serneels, 2008). Note that, like the salesforce compensation literature, this research stream investigates only a few types of experiences (e.g., firm or labor-market experience) and rarely the experiences of sales employees.

### Work experience research

In contrast to the two research streams above, the literature on *work experience* in industrial/organizational psychology and human resource management (column C in Table 1) investigates a much wider range of dimensions implicit in work experience. In a review of this research area, Quińones (2004) underscores the importance of models that consider the multifaceted nature of the construct of experience (i.e., Quińones et al., 1995; Tesluk & Jacobs, 1998). Combined, these frameworks encompass a richer set of measurement modes (i.e., time, amount, type, density or developmental impact, and timing) and levels of specificity (i.e., task, job, organization, work group, organizational level) than found in the other research streams. Although this literature highlights various work-related outcomes of work experience such as job performance (e.g., Beus et al., 2014; Huckman & Pisano, 2006; Van Iddekinge et al., 2019), leader performance (e.g., Avery et al., 2003; Sieweke & Zhao, 2015), or startup survival (e.g., Honoré, 2022), few published articles elaborate on the relationship between work experience and compensation (Werner & Ward, 2004). This dearth of research echoes Gupta and Shaw’s (2014) claim that compensation is a neglected area of human resource management research. Finally, in this strand of literature, research methods rarely control for individual heterogeneity.

### Career research

An extensive literature on *careers* (column D in Table 1) reflects multiple perspectives such as career growth (e.g., Liu et al., 2010), career advancement (e.g., Bowles et al., 2019), career success (for a review see Chen et al., 2021), protean and boundaryless career orientations (for a meta-analysis see Li et al., 2022), and influence of career stages on motivation (e.g., Cron et al., 1988). Notably, research investigating the relationship between career and compensation essentially examines internal and external career mobility (e.g., Bidwell, 2011; Bidwell & Mollick, 2015). But discussions and examples of career mobility tend to be limited to a few dimensions of work experience (i.e., number of job switches or firm or labor-market experience). In addition, in most cases, the current methodological practice falls short of completely controlling for individual heterogeneity. Finally, sales jobs are generally absent from this stream of research.

Drawing from the integrated insights contributed by the above four streams of research, we develop predictions regarding the impact of work experience on compensation. In keeping with Quinones’ (2004) and Tesluk and Jacobs’ (1998) conceptual frameworks (column C of Table 1), we study several facets of work experience that capture unique aspects of a salesperson’s career. We define relevant experience in terms of *time spent* working in a particular *firm*, *industry*, and *occupation* (i.e., *sales*) and at different *hierarchical levels* (i.e., *salesperson* or *sales manager*). As previously noted, none of the four strands of literature presented above examine these relevant facets of experience on compensation, let alone in a sales setting. Yet, prior research has reported increasing mobility of employees spanning firms, industries, and occupations (Bidwell & Mollick, 2015) and high turnover rates for salespeople (Boles et al., 2012). The current paper addresses this gap by investigating how distinct types of experience affect the compensation of salespeople and sales managers. We draw on Becker's (1962) widely known discussion of seniority–wage concave profiles that echoes the increased use of curvilinear U‐ and inverted U‐shaped relationships in strategy and, more generally, in management research (Haans et al., 2016).

## Hypotheses development

### How do multi-tier sales organizations value firm, industry, or sales occupation experience?

#### Benefits associated with salespeople’s firm, industry, or sales occupation experience

Salespeople can improve their selling skills throughout their career. As salespeople develop richer and more overlapping knowledge structures about customer types, their effectiveness increases (Sujan et al., 1988). Also, firm, industry, or sales occupation experience helps salespeople learn to identify behavioral cues and link them to customers' needs (Hall et al., 2015). This often results in better performance over time. These findings are consistent with Vosgerau et al. (2008), who show that perceptions of the quality of customer relationships improve in accuracy over time.

In addition, throughout their career, salespeople build skills such as technical product expertise and deep knowledge of buyer and seller organizations (Bradford et al., 2010; Broschak, 2004); and they develop social capital stemming from their nexus of relationships (Nahapiet & Ghoshal, 1998). This combination of knowledge, skills, and social capital makes salespeople increasingly valuable, especially when customer loyalty is a crucial firm asset and relational ties with salespeople are determinant. Therefore, a corollary is that firms’ benefits increase with a salesperson’s experience within a firm (i.e., intra-firm and buyers’ organizational networks to leverage internal resources, offering technical knowledge), an industry (i.e., cross- and within- industry relationships and knowledge to anticipate competitive dynamics), or sales occupations (i.e., selling skills, assessing customer’s and stakeholder’s profiles and relationship quality). Over the long term, however, these benefits become increasingly weaker, as knowledge and networks tend to become redundant and selling effectiveness more difficult to improve.

#### Costs associated with salespeople’s firm, industry, or sales occupation experience

Whereas a firm’s benefits stemming from salespeople’s experience increase at a decreasing rate and eventually level off, its costs continue to rise at an increasing rate. We theorize that two mechanisms explain the curvilinear shape of the cost function. The first mechanism, “technological evolution in the selling environment,” accounts for the effects of updating costs of knowledge and skills. Hence, evolving sales environments drive the need for personal selling adaptation across firm, industry, or sales occupation experience (Cron, 2017; Jones et al., 2005). As much research suggests, older and more experienced salespeople tend to have more negative perceptions about new technologies and may use them less frequently (Morris & Venkatesh, 2000; Speier & Venkatesh, 2002). That is to say that salespeople’s difficulty with or indifference to developing new abilities increases over time.

The second mechanism, “achievement weariness,” is the lack of motivation to keep up to date with changes in a firm's developments, industry shifts, and evolution of sales occupations. Salespeople’s lack of motivation for further performance improvements and unwillingness to expend effort typically increase as time passes (Cron & Slocum, 1986; Kanfer & Ackerman, 2004). Firms will underperform if their salespeople fail to improve and develop their skills, renew their knowledge, and enrich their professional networks. Costs related to professional obsolescence, therefore, rise at an increasing rate. In essence, firms are constrained to invest more (e.g., training, hiring younger salespeople or salespeople from another firm) to reach their business objectives when their salespeople become less capable. This may be partially due to the perception that developing new skills is more difficult and costly for high-seniority employees.

Together, these mechanisms drive professional obsolescence. With a growing mismatch of their salesforce, firms’ costs can increase at an increasing rate with salespeople’s experience.

As explained earlier, we combine the above latent benefit and cost functions additively to theorize an inverted U-shaped relationship between salespeople’s firm, industry, or sales occupation experience and compensation. Web Appendix A1 provides an illustration of this mechanism. Thus, we propose the following hypothesis:

##### H1

The relationships between (a) firm, (b) industry, or (c) sales occupation experience and compensation have an inverted U-shape for salespeople.

### Heterogeneity of experiences: Firm and industry experience

#### Benefits associated with firm and industry experience

Given that firms typically view *sales* roles as strategic, we suggest that this *"sales identity"* will signal *sales* competency and will drive a compensation premium because firms, unsure whether sales personnel with diverse work experiences are multi-competent or incompetent, are likely to opt for the safest path and value a focused *sales* career history (Ferguson & Hasan, 2013; Zuckerman et al., 2003). Furthermore, given that firms often have no other indicator of *sales* skills than employees’ track record (i.e., *firm*, *industry*, and *sales occupation* experience), employees’ *sales* experience will govern firms’ compensation decision *more than* their *firm* or *industry* experience.

In addition, we argue that *sales occupation* specificities call for skill sets rewarded at higher rates than other skill sets developed through *industry* or *firm* experience. This is because skills developed through *sales* experience are more general (i.e., transferable across firms and industries) than *industry*- or *firm*-specific skills, thereby more likely to be valued by external employers (Bernhardt & Timmis, 1990). In essence, salespeople’s abilities to characterize customers and stakeholders, understand buying organizations, perceive relationship quality, and develop social capital transcend firms’ and industries’ boundaries. By a logic similar to the one presented above, we speculate that the knowledge, skills, and social capital of salespeople increase at an increasing rate with their sales experience and eventually level off.

#### Costs associated with firm and industry experience

Given that the knowledge and skills developed through *sales* experience are more general (i.e., transferable across firms and industries) than *industry*- or *firm*-specific skills, we expect that their costs will rise more sharply over time than the costs related to *industry* or *firm* experience. This is because the updating resources have a wider, cross-industry and cross-firm focus. In essence, salespeople are motivated by the prospect of success to continuously update their knowledge and skills on all fronts. Therefore, the costs related to updating *sales*-related skills and knowledge are higher than the costs related to updating *industry*- or *firm*-related skills and knowledge.

All in all, we conclude that firms will reward sales occupation experience at higher rates than industry or firm experience. Web Appendix A2 illustrates this argument. Thus, we hypothesize the following:

##### H2a

The inverted U-shaped relationship between sales occupation experience and salespeople's compensation shifts upwards compared to that of industry experience.

##### H2b

The inverted U-shaped relationship between sales occupation experience and salespeople's compensation shifts upwards compared to that of firm experience.

### Moderating effect: Job position

#### Benefits associated with sales managers’ sales occupation experience

Scrutiny of our data on sales managers indicates that their sales occupation experience impacts compensation differently than for salespeople. Given that sales managers' opportunity cost of time is higher than that of salespeople—because effective sales managers have a multiplier effect on performance through their impact on subordinates (MacKenzie et al., 1998)—their compensation should increase at a higher rate than that of salespeople as their sales occupation experience increases. In essence, the impact of sales managers is derived from the way they develop their salespeople, each of whom may generate higher levels of sales. Thus, we argue that sales managers’ benefit function is steeper than that of salespeople. In a nutshell, sales managers have a multiplier effect that justifies paying them at higher rates than salespeople (Rouziès et al., 2009).

Furthermore, sales managers, who are generally responsible for strategy implementation and salesforce management, develop managerial skills and technical knowledge with sales job experience. Typically, they align their sales team's efforts with their firm's strategy through downward and upward influences (Ahearne et al., 2014). Over time, they accrue relationship-specific skills as they build social networks encompassing multiple hierarchical levels (Ahearne et al., 2013) and various stakeholders. Simultaneously, they develop knowledge about products and services, procedures, routines, employees, customers, and stakeholders. This knowledge, coupled with these specific social skills, is likely to become increasingly valuable as their quality improves. Eventually, however, administrative tasks linked to their managerial responsibilities leave them less and less time to upskill. Therefore, we expect sales managers’ benefits to increasingly weaken with higher levels of sales experience.

#### Costs associated with sales managers’ sales occupation experience

We argue that sales managers’ cost function is similar to that of salespeople. As explained earlier, “technological evolution” results in rising costs for firms. Like salespeople, sales managers have to update their knowledge and skills. Hence, evolving sales environments drive the need for personal selling adaptation (Cron, 2017; Jones et al., 2005). Sales managers need to integrate and analyze competitive intelligence collected by their salesforce to identify major trends and competitive forces impacting their markets. For that purpose, they tend to rely on high-quality information obtained from other expert sources (e.g., consultancies, etc.) (Pazy, 1990). Despite the fact that sales managers have to stay current on more fronts and through many more channels than salespeople, we argue that a firm’s investments to upskill managers are similar to investments in their salesforce because (1) managers generally do not collect the information themselves, and (2) they usually have access to more efficient updating resources. Overall, firms need to increasingly invest to improve the expertise of their more experienced sales managers and salespeople (or replace them with cheaper alternatives).

The above latent benefit and cost functions combine additively as shown in Web Appendix A3. We therefore hypothesize that the inverted U-shaped relationship between sales occupation experience and compensation is shifted to the right for sales managers as compared to salespeople. Therefore, we posit:

##### H3

With increasing levels of sales job positions, the inverted U-shaped relationship between sales occupation experience and compensation shifts to the right.

## Methods

### Data

We assembled data from a large-scale administrative database (DADS, 2022) based upon mandatory employers' reports of each employee's gross earnings subject to payroll taxes from 1994 through 2015 in France. From these reports of matched employers–employees, the National Institute of Statistics and Economic Studies of France (INSEE) compiles a panel of all employees born in October in even-numbered years (excluding civil servants). Essentially, every employee is entered into the database when they start working and is followed until they exit the labor market or till the end of the data-collection year. An observation in this initial DADS data includes an identifier corresponding to the employee (i.e., *ID*) and an identifier corresponding to the firm (i.e., *SIREN*). Each combination of *ID*-year-*SIREN* corresponds to an observation in the data. This means that for every year that an employee works, there is at least one observation. Moreover, every time the employee switches firms, another observation is generated. Thus, for each observation, we have information on the number of days and hours during the calendar year the individual worked in the firm, their gender, date and place of birth, occupation, total net earnings, and the location and industry of the employer.

We selected sales employees from the initial dataset using twenty-seven salespeople’s and eight sales managers’ four-digit occupation codes (PCS Professions et Catégories Socioprofessionnelles, INSEE) within the manufacturing sector that is identified through twenty two-digit industry codes (NACE rev.2, 2022), thereby generating a randomly selected sample of the salespeople’s population. Note that all the observations of individuals are sampled, even if the individuals worked only once (i.e., for one observation) as a sales employee between 1994 and 2015. Next, we retain all the observations corresponding to full-time employment with a duration of more than three months. Finally, after the main experience measures are constructed, as described in the measures section, the observations from the same individuals but not in the relevant jobs (i.e., not sales) are deleted. Additionally, to include a meaningful measure of experience, we considered individuals with more than two years of total experience and one year of sales experience. Hence all individuals had at least two observations as sales personnel. The final sample consists of 5,299 sales managers and 19,606 salespeople, resulting in 21,994 sales managers- and 79,380 salespeople-year-firm observations. More details on data compilation can be found in Web Appendix B.

### Measures

#### Compensation

To construct this dependent-variable measure, we use the log of the employee’s net total compensation in keeping with prior research in economics (Buchinsky et al., 2010). Net total compensation includes all types of direct and indirect payments, such as wages, incentives, bonuses, and other benefits that sales employees receive after tax. One other widely used dependent variable is the log of hourly compensation (i.e., net total compensation divided by the number of hours worked by the individual in a job spell). However, in light of the recent criticism of using ratio variables (Certo et al., 2020), we use the log of total net compensation and include the number of hours worked in that observation as a control variable.^2^ The results of both dependent variables are broadly consistent.

#### Experience

The independent variables (i.e., sales occupation, firm, and industry experience) are the aggregation of days worked in each sales job, firm, and industry from the date that a worker is first employed (i.e., the first observation of the individual) until the respective time of the observation. To obtain this measure by year, we divide the number of days by 360 (a full year of work is assigned 360 days in the dataset).

Our measure, *occupation experience,* is the total number of years that an individual accumulated in sales jobs from the beginning of their career until the observation, regardless of the firm or industry. Note that to construct this measure, we treat sales management and salespeople’s jobs as separate sales occupations.^3^ Likewise, *firm experience* is the total number of years that an individual worked in the current firm (the focal observation’s listed firm) until that observation, regardless of the occupation or industry. *Industry experience* is the total number of years an individual worked in the current two-digit industry (the focal observation’s industry) until that observation, regardless of the firm or occupation.

For example, consider an individual who entered the dataset in 1999 and worked until 2009, when she was a salesperson at the time. The total occupation experience for this salesperson in 2009 is the aggregation of the days she worked in sales jobs over 1999–2009 divided by 360. If the employee had two spells as a salesperson until 2009, namely 1999–2002 and 2005–2009, her sales occupation experience in 2009 is the sum of the days she worked during those two spells regardless of the firm or industry. This index creates a career history measure for each observation consisting of the occupation, firm, and industry experience. To account for the non-linearity of the relationship between different experiences and compensation, we also include the *quadratic terms* of each measure of experience.

#### Control variables

In addition to experience specific to a firm, occupation, and industry, the overall labor-market experience may affect compensation. For example, a salesperson may have started to work as a waiter in the service industry and then changed after a few years to become a salesperson. The experience gained in the restaurant is not captured in any of the independent variables but may affect her compensation. We operationalize *total work experience* as the difference between the year of the observation and the first year the worker entered the labor market. We also deduct the individual’s inactive periods from the final measure.

Another type of experience that can substantially impact sales managers' compensation is whether they have prior *non-sales managerial experience*, which can provide knowledge and skills to manage salespeople, thereby affecting the returns to sales management occupation experience. This variable is precisely constructed like the other types of experience with the caveat that it stops accumulating when an individual becomes a sales manager. In essence, it is measured before sales managers are observed in the sample. Therefore, it is time-invariant during the period that individuals are observed as sales managers. As mentioned above (i.e., in the description of the compensation variable measure), we also control for the *number of hours worked* by an individual during the given observation and for *education,* as empirical evidence exists on the latter’s positive effect on pay (Coughlan & Narasimhan, 1992). Only 12% of our initial sample included an objective measure of education that is collected through a supplementary survey called EDP ("Permanent Demographic Sample EDP" 2022).

EDP is a large-scale socio-demographic panel established in France to study the birth rate, mortality, relationships, geographical migrations, and social and professional mobility. The sample is selected randomly, based solely on a person’s date of birth, and corresponds broadly to a survey of 1% of the population (4 dates of birth in the year) before December 2006 and 4% after (16 dates of birth). EDP is matched to the DADS Panel we are using for this research. EDP measures education by the highest degree obtained by individuals according to eight-degree categories*.* We adapted the method used by Abowd et al. (1999) to estimate the level of education for the remainder of individuals.

We ran a multinomial logit model using these eight education categories as the dependent variable and data available in the sample. We used the same data and the estimated coefficients from this model to impute the category with the highest probability among the eight distinct categories for the individuals who were not part of the 12% EDP subsample. The actual values were used for the EDP sample members.

Because prior research suggests a relationship exists between sales employees’ pay and firm size (John & Weitz, 1989; Lo et al., 2011; Rouziès et al., 2009), *business unit size* is operationally defined as the number of employees in a business unit. Given that sales employees are often scattered across different independent units, and local compensation levels influence compensation policies, we use business unit size rather than overall firm size as a control variable. Our data specify the units of each firm using a separate coding scheme. Hence, we can identify the unit where each individual works.

Further, we control for *gender,* as pay inequalities are found in the workforce. Gender is a dummy variable that takes the value of 1 for male and 0 female. Because state-owned companies are under scrutiny for their labor policies, we include a dummy variable for *state-owned firm* that takes the value of 1 when the firm is state-owned and 0 otherwise. We include the *occupational concentration* in a region of a given occupation, i.e., salespeople and sales managers, to control for competition for employees and labor-market conditions. Occupational concentration is measured as the total number of employees in a given occupation in a region normalized by the size of the region in square kilometers. We also include *year dummies* to account for changes in compensation due to seasonal effects such as economic fluctuations. Finally, as the compensation norm within a group of organizations competing within industries is likely to affect the compensation level, we include 19 *industry dummies* that correspond to manufacturing sectors. The dummies are based on two-digit NACE rev.2 industry codes. A complete list of variables is included in Web Appendix C.

### Estimation procedure

To test our hypotheses, we estimate the parameters of the following baseline model for employee i, working at firm j, in job q, in industry d, at time t:

1 $$\begin{array}{c}\mathrm{ln}\;(\mathrm{Cijqt})={\upbeta }_{0}+{\upbeta }_{1}\;\mathrm{Firm}\_{\mathrm{Exp}}_{\mathrm{ijt}}+{\upbeta }_{2}\;\mathrm{ Industry}\_{\mathrm{Exp}}_{\mathrm{ijt}}+{\upbeta }_{3}\;\mathrm{ Occupation}\_{\mathrm{Exp}}_{\mathrm{iqt}}\\ +{\upbeta }_{4}\;\mathrm{ Work}\_ {\mathrm{Exp}}_{\mathrm{it}}+{\upbeta }_{5}\;\mathrm{ Managerial}\_{\mathrm{Exp}}_{\mathrm{it}}+\upgamma {\mathrm{X}}_{\mathrm{it}}+\updelta {\mathrm{Z}}_{\mathrm{i}}+{\uppsi }_{\mathrm{ij}}+{\upmu }_{\mathrm{iq}}+{\uplambda }_{\mathrm{ij}}+{\upvarepsilon }_{\mathrm{i}}+{\upvarepsilon }_{\mathrm{ijqt}},\end{array}$$

where ln (C_ijqt_) stands for the log of compensation for employee i, at firm j, in job q, and at time t; Firm_Exp_ijt_ represents the firm experience; and Industry_Exp_ijt_ and Occupation_Exp_iqt_ represent the experience in the current industry, and occupation, respectively. Please note that the subscript d is not used because the industry is the same as the one that firm j belongs to. Thus, the subscript j replaces d in the equation. Quadratic terms of all experience measures are also included in the regressions but omitted in the model statement for ease of exposition. Work_Exp_it_ represents the overall labor-market experience. Managerial_Exp_it_ represents the prior non-sales managerial experience that is time-invariant over a sales manager's job spell as sales manager. Vector X_it_ includes time-varying explanatory variables such as industry, occupation indicators, and year indicators. Z_i_ is a vector of time-invariant explanatory variables such as gender. The individual’s inherent ability and motivation are captured by the individual fixed effect *ε*_*i*_. In addition, random variations in wages that are independent across time are captured by ε_ijqt_.

### Endogeneity concerns and instrumental variables

To estimate the above model, and in particular, the various returns to experience in the sales profession, we need to address potential endogeneity of these variables. In this type of marketing research, as recently pointed out by Rutz and Watson (2019), we must account for three important sources of endogeneity. First, the right-hand-side variables of interest—the different returns to experience—are likely to be correlated with earnings because both are likely to depend on some unobserved individual-specific common factor, such as dynamism, that might explain both performance (and therefore high wages) and a tendency to move relatively rapidly between jobs and/or firms, hence affecting mobility and our measures of experience. Both are also likely to depend on some common unobserved match-specific factor. This is the *simultaneity* issue outlined by Rutz and Watson (2019). These correlations would clearly bias the estimates, even when controlling for individual and match-specific fixed effects (the *omitted variable* bias, using these authors’ categorization). This type of endogeneity (*simultaneity*) was Altonji and Shakotko’s (1987) main concern. It will be discussed below and addressed using an IV strategy that we describe later, directly borrowed from these authors’ contributions. *Measurement error* in the independent variables could also bias our estimates (Rutz and Watson’s (2019) third category). However, we believe this concern is less urgent given the fiscal (administrative) nature of our data source from which we measure earnings and experience, including firm-specific experience.

To be more explicit about the sources of simultaneity: individuals’ ability and motivation will both affect compensation and experience. The first component of an *individual’s inherent ability and motivation* is likely to be independent of the firm, industry, and occupation—for instance, integrity, cognitive ability (Cron et al., 2005), and intelligence (Verbeke et al., 2008). We would expect a positive correlation between this component and firm, industry, and occupation experience, as high-performing salespeople or sales managers could be both more likely to receive higher compensation and less likely to quit or be laid off, thereby increasing their firm experience and compensation at the same time. However, their reputation could induce outside job offers and, thereby, decrease their firm experience. Although less clear, there may be a similar correlation between this first component and the length of industry and sales occupation experience. High performers may prefer to stay in a sales occupation longer while simultaneously commanding higher compensation.

The second component is *the match between an individual’s abilities and motivation and firm characteristics*. This unobserved match value between the salesperson and the firm is denoted as ψ_ij_ (Jovanovic, 1979; Parsons, 1986). An example of firm match value could be the match between salespeople’s personality traits and the control system of the organization (Miao & Evans, 2012) or salesforce structure, such as key account management (KAM) systems (Guenzi et al., 2007). We expect a positive correlation between firm–individual match value with both the length of firm experience and compensation.

*The third unobserved component is the match between an individual’s abilities and motivation and the industry’s characteristics,* denoted as λ_ij_. An example could be the fit between salespersons’ educational background and the industry’s customer base (Jaworski, 1988). This industry–individual match component is likely to increase both the length of industry experience and the compensation of individuals.

Finally*, the unobserved match between individuals’ abilities and motivation and the sales occupation might be correlated with both occupation experience and compensation*. For example, individuals with certain traits like extroversion (Vinchur et al., 1998) or greater cognitive aptitude (Verbeke et al., 2011) are a better fit for sales jobs that, in turn, will positively affect both the length of occupation experience and compensation. These match values are unobserved but are considered by salespeople and sales managers when they make employment choices. Further, this model of compensation implies that employees will self-select into firms, industries, and sales occupations based on their innate ability and motivation.

To address these concerns, we use an instrumental-variable strategy directly adapted from Altonji and Shakotko (1987).^4^ For the simultaneity issue, this procedure develops instruments for current firm experience with the deviations of current experience at the firm from the mean of firm experience for that person. Suppose Firm_Exp_ijt_ is the firm experience of individual i, with firm j, in period t, and $\overline{{\mathrm{Firm}\_\mathrm{Exp}}_{\mathrm{ij}}}$ is the average tenure of individual i, during the current spell of working with firm j. In that case, the instrumental variable for firm experience is:

2 $$\mathrm{Inst}(\mathrm{Firm}\_{\mathrm{Exp}}_{\mathrm{ijt}})=\mathrm{Firm}\_{\mathrm{Exp}}_{\mathrm{ijt}}-\overline{{\mathrm{Firm}\_\mathrm{Exp}}_{\mathrm{ij}}}$$

Notice that *ε*_*i*_ and ψ_ij_, representing the time-invariant individual fixed effect and firm–individual match effects over the period of the individual’s tenure with the firm, respectively, are constant over the duration of experience in firm j. Inst(Firm_Exp_ijt_) sums up to zero over the years that individual i, is with firm j, and the subtraction of $\overline{{\mathrm{Firm}\_\mathrm{Exp}}_{\mathrm{ij}}}$ from Firm_Exp_ijt_ cancels out the component of experience that is correlated with ε_i_ and ψ_ij._ for each observation. Hence, the instrument by construction is uncorrelated with the error terms, satisfying the exclusion (exogeneity) restriction. We construct instruments for occupation and industry experience and their quadratic terms, as well as their interactions with sales-manager indicators in the same manner. As stated, the instruments for the match values are uncorrelated with the time-invariant unobserved individual heterogeneities that affect the performance (and the associated earnings) of individuals in a firm, occupation, and industry over the period that the individual stays in a given firm, or industry, and occupation. This method creates one IV per endogenous variable,^5^ which means the equations are exactly identified, preventing us from using formal over-identification tests. However, formal statistical tests are conducted with the help of additional instruments to confirm instruments’ relevance and exogeneity, as explained below.

To complement the main instruments discussed above, we construct heteroskedasticity-based instrumental variables (HBIV) (Lewbel, 2012).^6^ In fact, Baum and Lewbel (2019) argue that the best use of HBIVs is in conjunction with external instruments. That way, over-identification and application of Sargan–Hansen tests can be used. This would not be possible where regression equations are exactly identified. The results of the models with and without constructed instruments are widely consistent. We report the results of models that combine HBIV and external instruments in Tables 4 and 5. Kleibergen and Paap (2006) rank Wald F-tests for weak identification of the instrumental variables are above the 10% critical value of 16.38, which proves the strength of the instruments (as suggested by Stock and colleagues (2002)). We used Hansen’s J-statistic of over-identifying restrictions (Hansen, 1982) and C-statistic (difference in Sargen test) (Hayashi, 2011) for the exogeneity of each instrument separately. As expected, we were unable to reject the null hypotheses that our external (deviations from the mean of experience) instruments are uncorrelated with the error terms.

### Multi-collinearity, identification, and other econometric adjustments

Multi-collinearity is an often-cited problem when estimating regression models that include multiple experience variables that increase by one every year unless they are reset to zero after a mobility episode. It is also present when one includes the quadratic terms of these experience variables, as in our model. Even though multi-collinearity appears of less concern to econometricians in recent works, in our setting, it is essentially equivalent to a question central to modern econometrics: *identification.* In our case, ensuring *separate identification* of the different returns to experience is important. Indeed, mobility between firms, between occupations including from salesperson to sales manager, and between industries must be sufficiently active for such separate identification to be achieved. The mobility statistics of salespeople and sales managers between firms, industries, and occupations are presented in Web Appendix D1-D4. Our examination of multi-collinearity will also allow us to alleviate these identification concerns.

Focusing on classical methods, the variance inflation factor (VIF) may reproduce non-essential multi-collinearity (Robins et al., 2009). Hence, we follow a sequential residual centering method (Francoeur, 2013; Singh et al., 2018). As mentioned above, the issue is most pressing for our experience variables. Therefore, we focus on them in our analysis.

When the quadratic and the interaction of the sales manager’s dummy with the experience variables are orthogonalized through residual centering, the VIFs are lower than 10, suggesting that collinearity is not a threat to the validity of the results (Belsley et al., 1980; Marquaridt, 1970).

To help determine whether our model estimates are affected by outliers, we winsorize continuous variables at the 1st and 99th percentile (Kurt & Hulland, 2013). Generally, outliers are not an issue for experience variables because we did not observe extreme values for these measures. However, the winsorization of compensation at the 1^st^ and 99^th^ percentile did not affect our results. To ensure robustness to autocorrelation and heteroskedasticity, we ran our main instrumental-variable method computing standard errors adjusted to heteroskedasticity and autocorrelation (HAC) using a weighting matrix with a Bartlett (Newey-West) kernel (Driscoll & Kraay, 1998; Tavassoli et al., 2014) and also adjusted for clustering at the firm level (Thompson, 2011). Again, results were unaffected.

## Results

In Tables 2 and 3, we present the descriptive statistics and the correlation coefficients between the variables of interest for salespeople and sales managers, respectively. The results of the 2-stage least-squares (2SLS) analyses of sales managers (Model 2) and salespeople (Model 4) appear in Table 4. Table 5 presents the results of the 2SLS models that test our hypotheses for the pooled salespeople's and sales managers' samples. First, we create a dummy "SM" with a value of 1 for sales managers and 0 for the salespeople in the pooled salespeople's and sales managers' data. We then add interaction terms between this dummy and all the variables of interest (i.e., industry, firm, or sales occupation experience) to Eq. 1 and re-estimate the equations (Models 2, 3, and 4 for industry, firm, or sales occupation experience, respectively). In addition, we include a control variable to account for sales managers’ previous non-sales managerial experience (measured *before* sales managers are observed in the sample).^7^ The results of the estimations show that the interactions between the sales manager dummy (SM dummy) and the firm (Model 2) and industry (Model 3) variables are significant for both linear and quadratic terms. However, the interaction between the SM dummy and the sales occupation experience variables is only significant for the linear term (Model 4). Further, the results confirm the hypotheses, as explained below.

**Table 2:** Correlation coefficients and summary statistics for salespeople

		Mean	S.d	1	2	3	4	5	6	7	8	9	10
1	Net Total Compensation^a^	9.68	.592	1									
2	Industry Experience	4.35	3.92	.362	1								
3	Firm Experience	3.82	3.51	.427	.763	1							
4	Sales Occupation Experience	4.99	4.23	.394	.521	.549	1						
5	Hours Worked	1,505	563	.637	.302	.374	.296	1					
6	State-Owned Company	.001	.015	-.009	-.009	-.006	-.012	-.010	1				
7	Gender	.584	.492	.171	.064	.053	-.008	.051	-.006	1			
8	Total Work Experience	8.34	3.51	.316	.445	.411	.350	.229	-.004	.068	1		
9	Business Unit Size	171	618	.082	.004	.020	.077	.032	.001	.020	.019	1	
10	Occupational Concentration	1.00	1.22	.072	-.029	-.014	.042	.017	-.004	-.129	.048	.031	1

^a^This variable is measured as a log

Correlations greater than or equal to .001 are significant at *p* < *.01*

**Table 3:** Correlation coefficients and summary statistics for sales managers

		Mean	S.d	1	2	3	4	5	6	7	8	9	10	11
1	Net Total Compensation^a^	10.3	.539	1										
2	Industry Experience	5.18	4.37	.374	1									
3	Firm Experience	4.49	3.87	.419	.708	1								
4	Sales Occupation Experience	5.16	4.26	.422	.453	.491	1							
5	Hours Worked	1,667	440	.706	.263	.345	.271	1						
6	State-Owned Company	.001	.025	-.003	.033	.043	.018	.021	1					
7	Gender	.726	.446	.114	.083	.068	.071	.023	-.041	1				
8	Total Work Experience	9.56	3.65	.334	.421	.383	.333	.112	.003	.086	1			
9	Prior Managerial Experience	1.72	2.96	.217	.086	.068	-.028	.064	-.009	-.022	.021	1		
10	Business Unit Size	226	471	.081	-.003	.014	.018	.040	.025	-.058	-.037	.123	1	
11	Occupational Concentration	1.22	1.24	.101	-.043	-.032	.007	.003	-.010	-.137	.032	.093	.032	1

^a^This variable is measured as a log

Correlations greater than or equal to .001 are significant at *p* < *.01*

**Table 4:** Two stage least square regressions for sales personnel (including combined instrumental variables)

	Sales Managers^a^		Salespeople^b^	
SM and SP	Model 1	Model 2	Model 3	Model 4
Variable	Baseline	Combined IV	Baseline	Combined IV
Firm Experience		0.018*** (0.001)		0.007*** (0.001)
Firm. Experience^2^		-0.003*** (2.6E-4)		-0.001*** (2.7E-4)
Industry Experience		0.010*** (0.001)		0.004*** (0.001)
Industry Experience^2^		-0.002*** (2.2E-4)		-0.001** (2.5E-4)
Sales Occupation Exp		0.042*** (0.001)		0.019*** (0.001)
Sales Occupation Exp.^2^		-0.003*** (3.8E-4)		-0.003*** (3.1E-4)
Total Work Experience	0.032*** (0.001)	0.020*** (0.001)	0.025*** (4.7E-4)	0.022*** (4.6E-4)
Non-Sales Managerial Exp	0.020*** (0.001)	0.024*** (0.001)		
Hours Worked	0.001*** (6.7E-6)	0.001*** (7.1E-6)	0.001*** (5.0E-6)	0.001*** (5.1E-5)
Gender	0.079*** (0.006)	0.072*** (0.005)	0.094*** (0.003)	0.096*** (0.003)
State-Owned Company	-0.416*** (0.052)	-0.493*** (0.049)	-0.115 (0.069)	-0.102 (0.069)
Business Unit Size	1.0E-4*** (6.3E-6)	1.0E-4*** (5.7E-6)	1.0E-4*** (2.4E-6)	1.0E-4*** (2.4E-6)
Occupational Concentration	0.032*** (0.002)	0.034*** (0.002)	0.016*** (0.001)	0.017*** (0.001)
Education Dummies	Yes	Yes	Yes	Yes
Year and Industry Dummies	Yes	Yes	Yes	Yes
Constant	8.986*** (0.085)	9.071*** (0.071)	8.416*** (0.029)	8.354*** (0.018)
Number of Observations	21,994	21,994	79,380	79,380
Number of Employees	5,299	5,299	19,606	19,606
Kleibergen-Paap Wald rk F		748		1,338
R^2^	0.630	0.670	0.580	0.586

^*^ *p* < 0.05, ** *p* < 0.01, *** *p* < 0.001

^a^ The dependent variable is the log of sales managers’ net total compensation

^b^ The dependent variable is the log of salespeople’s net total compensation; Heteroskedasticity and autocorrelation robust (HAC) estimation standard errors adjusted for clustering at firm level are reported in parentheses

**Table 5:** Two-stage least square regressions for sales personnel (pooled sample) (including combined instrumental variables)

Variable	Model 1 Baseline	Model 2 Combined IV	Model 3 Combined IV	Model 4 Combined IV
Firm Experience		0.016*** (0.001)	0.017*** (0.001)	0.017*** (0.001)
Firm. Experience^2^		-0.003*** (1.9E-4)	-0.003*** (1.9E-4)	-0.003*** (1.8E-4)
Industry Experience		0.005*** (0.001)	0.003*** (0.001)	0.005*** (0.001)
Industry Experience^2^		-0.001*** (2.1E-4)	-0.001** (2.2E-4)	-0.001*** (2.0E-4)
Sales Occupation Exp		0.023*** (0.001)	0.023*** (0.001)	0.020*** (0.001)
Sales Occupation Exp.^2^		-0.003*** (2.7E-4)	-0.003*** (2.7E-4)	-0.003*** (3.0E-4)
S.M. x Firm Experience		0.010*** (0.001)		
S.M. x Firm. Experience^2^		0.001* (2.6E-4)		
S.M. x Industry Experience			0.013*** (0.001)	
S.M. x Industry Experience^2^			-0.001* (2.7E-4)	
S.M. x Sales Occupation Exp				0.020*** (0.002)
S.M. x Sales Occupation Exp.^2^				-1.8E-5 (4.6E-4)
S.M	0.129*** (0.005)	0.140*** (0.005)	0.137*** (0.005)	0.135*** (0.005)
Total Work Experience	0.030*** (3.7E-4)	0.022*** (4.1E-4)	0.022*** (4.1E-4)	0.022*** (4.0E-4)
Non-Sales Managerial Exp	0.0258*** (0.001)	0.027*** (0.001)	0.026*** (0.001)	0.027*** (0.001)
Hours Worked	0.001*** (4.0E-6)	0.001*** (4.6E-6)	0.001*** (4.6E-6)	0.001*** (4.5E-6)
State-Owned Company	-0.247*** (0.051)	-0.264*** (0.057)	-0.268*** (0.057)	-0.261*** (0.056)
Gender	0.095*** (0.003)	0.095*** (0.003)	0.095*** (0.003)	0.094*** (0.003)
Business Unit Size	1.0E-4*** (2.5E-6)	1.0E-4*** (2.3E-6)	1.0E-4*** (2.3E-6)	1.0E-4*** (2.4E-6)
Occupational Concentration	0.019*** (0.001)	0.022*** (0.001)	0.022*** (0.001)	0.022*** (0.001)
Education Dummies	Yes	Yes	Yes	Yes
Year and Industry Dummies	Yes	Yes	Yes	Yes
Constant	8.554*** (0.019)	8.324*** (0.017)	8.32*** (0.018)	8.326*** (0.017)
Number of Observations	101,374	101,374	101,374	101,374
Number of Employees	24,900	24,900	24,900	24,900
Kleibergen-Paap Wald rk F		1,407	1,307	987
R^2^	0.621	0.659	0.658	0.659

^Ɨ^ *p* < 0.1, * *p* < 0.05, ** *p* < 0.01, ***p < 0.001

^a^ The dependent variable is the log of sales managers or salespeople's net total compensation

^b^ S.M. is a dummy variable (1 if sales manager, 0 otherwise); Heteroskedasticity and autocorrelation robust (HAC) estimation standard errors adjusted for clustering at firm level are reported in parentheses

Regarding the hypothesized inverted U-shaped functions between compensation and firm experience of salespeople (i.e., H1a) based on Haans and colleagues (2016), we first note that the coefficient of the quadratic firm experience in Model 4 (Table 4) is negative and significant (β = -0.001, *p* < 0.001). We also test the slope coefficients at the low (X_Firm_Exp_low_) and high (X_Firm_Exp_high_) ends of our data range (Haans et al., 2016). We find a positive and significant slope at the low end of the data range (b_low_ = β_Firm_Exp_ + β_Firm_Exp^2_X_Firm_exp_low_ = 0.010, *p* < 0.001) and a negative and significant slope at the high end of the data range (b_high_ = β_Firm_Exp_ + β_Firm_Exp^2_ X_Firm_exp_high_ = -0.059, *p* < 0.001). Additionally, the turning point lies within the observed data range [X_Firm_Exp_ = 2.9 years]. The satisfaction of the above three conditions lends support to H1a.

Regarding the hypothesized inverted U-shaped function between industry experience and compensation of salespeople (i.e., H1b), our analyses yield similar results. The quadratic term is negative and significant (β = -0.001, *p* < 0.01). Additionally, the slope at the low end of the data range is positive and significant (b_low_ = β_Industry_Exp_ + β_Industry_Exp^2_X_Industry_exp_low_ = 0.004, *p* < 0.001) and negative and significant at the high end of the data range (b_high_ = β_industry_Exp_ + β_industry_Exp^2_ X_industry_Exp_high_ = -0.026, *p* < 0.001). Finally, the turning point lies within the observed data range [X_Industry_Exp_ = 3.1 years]. All the above support the inverted U-shaped relationship hypothesized in H1b in keeping with Haans et al. (2016).

Finally, we find support for the inverted U-shaped form of the relationship between occupation experience and compensation for salespeople Thus, the quadratic term is negative and significant (β = -0.003, *p* < 0.001). Moreover, the slope at the low end of the data range is positive and significant (b_low_ = β_Occupation_Exp_ + β_Occupation_Exp^2_X_Occupation_Exp_low_ = 0.014, *p* < 0.001) and negative and significant at the high end of the data range (b_high_ = β_Occupation_Exp_ + β_Occupation_Exp^2_ X_Occupation_Exp_high_ = -0.101, *p* < 0.001). The turning point lies within the observed data range [X_Occupation_Exp_ = 3.4 years].

**Fig. 1:**
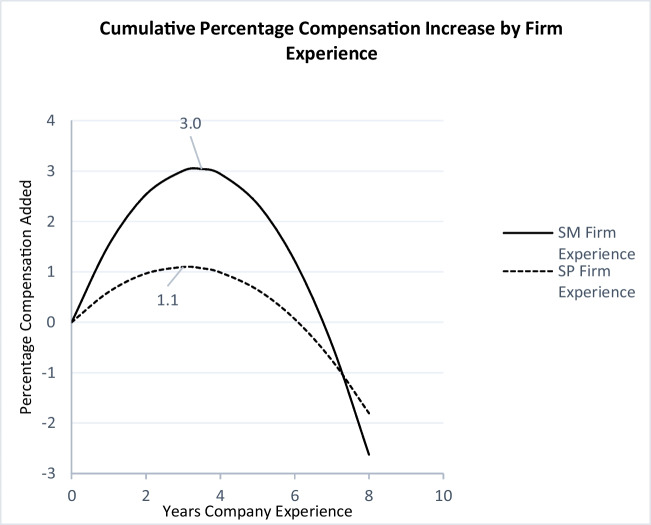
Firm experience and compensation Note: A salesperson on the dashed curve has 0 years of firm experience at the start of his/her career. After six years, his/her compensation has increased by 0.06% as a result of his/her cumulated firm experience.

**Fig. 2:**
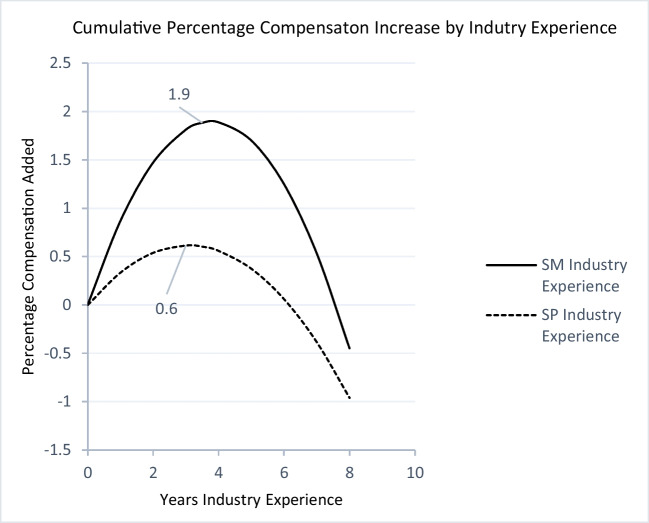
Industry experience and compensation Note: A salesperson on the dashed curve has 0 years of industry experience at the start of his/her career. After 6 years, his/her compensation has increased by 0.06% as the result of his/her cumulated industry occupation experience.

**Fig. 3:**
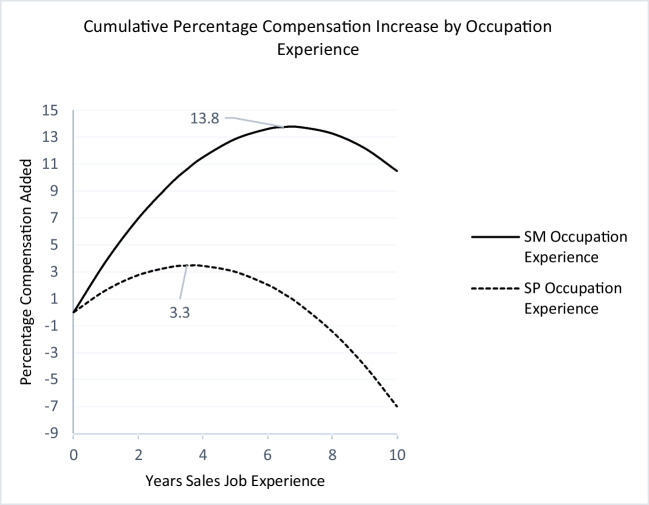
Occupation experience and compensation Note: A salesperson on the dashed curve has 0 years of sales occupation experience at the start of his/her career. After six years, his/her compensation has increased by 2% as the result of his/her cumulated sales occupation experience.

We perform similar tests (available upon request) and confirm the U-shaped relationships between firm, industry, and occupation experience and compensation at the sales-management level. The main regression model for sales managers is reported in Table 4 (Model 2). Finally, the functions between firm, industry, and occupation experience and compensation, for salespeople and sales managers, are presented in Figs. 1, 2 and 3.

To test H2a and H2b (i.e., an upward shift of compensation for sales occupation experience as compared to those for industry or firm experience for salespeople), we perform parameter comparison tests as suggested by Haans and colleagues (2016). A close examination of Model 2 coefficients (Table 4) shows that the coefficient of sales occupation experience^2^ ($\upbeta =-.0026)$ is significantly smaller than those of industry experience^2^ ($\upbeta =-.0007)$ or firm experience^2^ ($\upbeta =-.00012)$ for salespeople, providing support for H2a (*p* < 0.001) and H2b (*p* < 0.01), respectively. In sum, these results suggest that salespeople’s “sales identity” is the main driver of compensation for salespeople and sales managers.

To illustrate the impact of experience on compensation, we note that the compensation increases for sales managers (Model 2 in Table 4) and salespeople (Model 4 in Table 4) are 1.8% and 0.7%, respectively (535 and 112 Euros, respectively; calculations based on average net yearly compensation) with each year of experience within the same firm. The occupation experience coefficient for sales managers indicates that each additional year in sales-related occupation experience will add 4.2% (*p* < 0.001) to their compensation (1,250 Euros based on average yearly compensation), while this figure is only 1.9% (*p* < 0.001) for salespeople (304 Euros based on average yearly compensation). Furthermore, these values diminish over time: both groups' quadratic terms are negative (*p* < 0.001). The lowest return for both groups correspond to industry experience at 1% and 0.4% for sales managers and salespeople, respectively.

To test H3 (i.e., the moderating effect of job-position level), we first need to perform two derivatives of Eq. 3 (where Eq. 3 corresponds to Model 4 in Table 5).

3 $$\begin{array}{c}\mathrm{ln }(\mathrm{C})={\upbeta }_{0}+{\upbeta }_{\mathrm{Occupation}\_\mathrm{Exp}}\mathrm{ Occupation}\_\mathrm{Exp}+ {\upbeta }_{\mathrm{Occupation}\_\mathrm{Exp}{^\wedge}2}\mathrm{ Occupation}\_\mathrm{Exp}{^\wedge}2 +\\ {\upbeta }_{\mathrm{Occupation}\_\mathrm{Exp}\times \mathrm{SM\; Dummy}} (\mathrm{Occupation}\_\mathrm{Exp}\times \mathrm{SM\; Dummy})+{\upbeta }_{\mathrm{Occupation}\_\mathrm{Exp}{^\wedge}2\times \mathrm{ SM\; Dummy}}\\ (\mathrm{Occupation}\_\mathrm{Exp}{^\wedge}2 \times \mathrm{SM\; Dummy})+{\upbeta }_{\mathrm{SM\; Dummy}}\mathrm{ SM\; Dummy}+\mathrm{\delta X}\end{array}$$

Notably, the main variables of interest—SM Dummy, Occupation_Exp, Occupation_Exp^2, and the interaction of those experiences with the SM Dummy—are depicted separately in Eq. 3. The vector X represents the remainder of the variables.

We first derive Eq. 3 regarding occupation experience, leading to Eq. 4 below:

4 $${\mathrm{Occupation}\_\mathrm{Exp}}^{*}=\frac{{-\upbeta }_{\mathrm{Occupation}\_\mathrm{Exp}}- {\upbeta }_{{\mathrm{Occupation}\_\mathrm{Exp}}_{\times }\mathrm{ SM \;Dummy}} \, \times \mathrm{ SM \;Dummy} }{2\; {\upbeta }_{\mathrm{Occupation}\_\mathrm{Exp}{^\wedge}2}+2 \;{\upbeta }_{\mathrm{Occupation}\_\mathrm{Exp}{^\wedge}2 \times \mathrm{SM \;Dummy }}\times \mathrm{SM\; Dummy}}$$

Also, since Eq. 4 depends on the moderator (i.e., sales manager dummy: SM Dummy), we take its derivative to identify the direction of the turning-point shift (Haans et al., 2016), leading to Eq. 5 below:

5 $$\frac{\partial {\mathrm{ Occupation}\_\mathrm{Exp}}^{*}}{\partial \mathrm{SM\; Dummy}}=\frac{{(\upbeta }_{\mathrm{Occupation}\_\mathrm{Exp }}{\upbeta }_{\mathrm{Occupation}\_\mathrm{Exp}{^\wedge}2 \times \mathrm{SM\; Dummy}}- {\upbeta }_{\mathrm{Occupation}\_\mathrm{Exp}{^\wedge}2} {\upbeta }_{\mathrm{Occupation}\_\mathrm{Exp }\times \mathrm{ SM\; Dummy}})}{2{({\upbeta }_{\mathrm{Occupation}\_\mathrm{Exp}{^\wedge}2}+ {\upbeta }_{\mathrm{Occupation}\_\mathrm{Exp}{^\wedge}2 \times \mathrm{ SM\; Dummy}}\times \mathrm{SM\; Dummy})}^{2}}$$

Given that the denominator is always positive in Eq. 5, the direction of the shift in the turning point will only be determined by the numerator. Using parameters from Model 4 in Table 5, we observe that the numerator is positive, indicating a shift of turning point to the right ${(\upbeta }_{\mathrm{Occupation}\_\mathrm{Exp }}\;{\upbeta }_{\mathrm{Occupation}\_\mathrm{Exp}{^\wedge}2 \times \mathrm{SM\; Dummy}}- {\upbeta }_{\mathrm{Occupation}\_\mathrm{Exp}{^\wedge}2} \;{\upbeta }_{\mathrm{Occupation}\_\mathrm{Exp}\times \mathrm{ SM\; Dummy}}>0).$  

Based on the second derivative (Eq. 5), we test whether the shift is significant. We confirm that Eq. 5 is positive and significantly different from 0 at high (*p* < 0.01) and low (*p* < 0.001) levels of SM Dummy, providing support for H3. The turning point is 3.4 (*p* < 0.001) and 6.4 (*p* < 0.001) for salespeople and sales managers, respectively. The difference in turning points (∆ turning point) is three years (*p* < 0.001). Notably, we do not observe a significant effect of the interaction between the quadratic term of occupation experience and SM Dummy in Model 4 (Table 5), indicating that flattening or steepening does not occur in the case of sales-occupation experience (Haans et al., 2016; Vomberg et al., 2020). However, such an effect is evident in Model 2 (Table 5) for firm experience ($\upbeta$_S.M. x Firm. Exp^2_ = -0.001, *p* < 0.01) and Model 3 (Table 5) for industry experience ($\upbeta$_S.M. x Industry_Exp2_ = -0.001, *p* < 0.01), indicating steepening of the inverted U-shape for both curves at the sales-management level. Figures 1 and 2 depict these changes for firm experience and industry experience, respectively.

Not surprisingly, we find that gender inequality exists.: male sales managers and salespersons are paid 7.2% and 9.6%, respectively, more than their female counterparts (*p* < 0.001). Please note that we do not establish causality nor claim that the magnitude of coefficients for gender is accurate.

To assess confidence in the validity of the results and explore further implications, we run three additional analyses, as explained below. To examine a salesperson’s career progression to sales management, we first conduct a descriptive statistical analysis. This allows us to assess and contrast the compensation results with respect to career progression outcomes for salespeople with various experiences (firm industry and occupation). To grasp the respective steepness of salespeople’s and sales managers’ returns to occupation, firm, and industry experience, we then employ graphical analyses. Finally, we compare sales jobs to accounting and human resource (HR hereafter) jobs in terms of occupation, firm, and industry returns to experience.

### Mobility and career progression analysis

To gain insight into the career advancement of salespeople toward sales management roles, we conducted supplementary descriptive analyses. Web Appendix E reports statistics for salespeople with 2 to 9 years of sales-occupation experience to determine how their industry experience, firm experience, and compensation differ. The statistics in this table show mobility away from sales jobs through time (decreasing number of observations, first line). As sales experience increases by one year from column to column, the 75^th^ percentile of industry experience increases by more than one year. Thus, mobility appears to often occur *within* industry movements, as the 75^th^ percentile of industry experience shows. Given that the converse is true for the 25^th^ percentile of company experience, mobility may take place between firms (with the probability of a firm switch declining when sales experience increases, more so than an industry or occupation switch). Finally, the probability of becoming a sales manager decreases (from 0.22 to 0.16), while the time to becoming a sales manager increases with sales experience (from 7.88 years to 12.38 years, 0.35 years with an addition of one year to sales experience), indicating that an additional year of experience as a salesperson contributes little toward promotion.

Web Appendix F shows similar statistics at the time of promotion to sales management. First, promotion to sales management is more frequent for managers without sales experience (67% of newly promoted sales managers do not have any salesperson experience). Second, the two categories (i.e., with and without salesperson experience) tend to have different career trajectories, although they generally come from the same firm and industry. However, sales managers without sales experience have less firm, industry, and total work experience than those with sales experience. In terms of compensation, additional years of past salesperson experience bring little return at the moment of promotion; presumably, returns are expected to accrue later (during their time in a managerial position).

Given that experience in another managerial position is more likely for sales managers without sales experience (65% come directly from such a position) than for those with experience of sales (14% come directly from such a position), it is not surprising that they are better paid. Notably, the compensation difference between newly promoted sales managers with and without prior non-sales managerial experience (i.e., 13,329 Euros) is much larger than that of newly promoted sales managers with and without prior salesperson experience (i.e., 8,578 Euros).

Table 6 presents descriptive statistics for sales managers according to their occupation experience at the time of observation. Interestingly, the *increasing* proportion of sales managers with prior non-sales managerial experience across the columns of Table 6 indicates that they are more likely to stay in sales-management jobs. However, the *length* of prior non-sales managerial experience is relatively stable over all the categories, ranging from 2.47 to 2.24. This means that the *length* of prior managerial experience gives no indication on the length of sales-management jobs. Moreover, a comparison of industry and company experience across the columns of Table 6 shows that industry experience is generally greater than company experience: we can conclude that sales managers are more likely to stay in an industry while switching firms.

**Table 6:** Summary statistics on sales managers categorized by sales management experience

	Number of Years of Sales Management Experience							
	2 Years	3 Years	4 Years	5 Years	6 Years	7 Years	8 Years	9 Years
Number Observations	5,458	4,358	3,556	2,934	2,389	1,908	1,537	1,246
Proportion with Prior Salesperson Experience	0.32	0.31	0.29	0.27	0.26	0.23	0.21	0.19
Industry Experience	4.91	5.65	6.41	7.17	7.93	8.73	9.39	10.02
25 Centile	2.59	3.45	4.34	5.28	6.00	7.00	8.00	8.83
50 Centile	3.00	4.00	5.00	6.00	7.00	8.00	9.00	10.00
75 Centile	6.54	7.00	8.00	8.99	9.96	10.99	11.50	12.00
Company Experience	4.33	5.00	5.69	6.36	7.05	7.70	8.33	8.90
25 Centile	2.00	3.00	3.92	4.65	5.00	5.55	5.96	6.25
50 Centile	3.00	4.00	5.00	6.00	7.00	8.00	8.92	9.83
75 Centile	5.36	6.00	7.00	7.83	8.79	9.92	10.5	11.00
Sales Management Experience	1.98	2.98	3.98	4.98	5.97	6.97	7.97	8.97
Total Work Experience	12.58	13.51	14.38	15.21	16.13	16.93	17.67	18.13
25 Centile	7.00	8.00	9.00	10.00	11.00	12.00	13.00	14.00
50 Centile	11.00	12.00	12.00	13.00	14.00	15.00	16.00	16.00
75 Centile	16.00	16.00	17.00	18.00	19.00	19.00	20.00	20.00
Salesperson Experience at Promotion	4.31	3.99	3.87	3.73	3.61	3.33	3.19	2.79
Proportion with Prior Non-Sales Managerial Experience	0.54	0.56	0.58	0.60	0.63	0.65	0.65	0.66
Length of Prior Non-Sales Managerial Experience	2.47	2.38	2.36	2.29	2.28	2.36	2.35	2.24
Net Annual Compensation^1^	42,109	44,019	46,002	48,114	50,283	52,060	54,296	56,326
25 Centile	30,575	32,113	33,859	35,454	36,797	38,385	40,029	41,133
50 Centile	37,394	38,953	40,625	42,450	44,138	45,929	47,669	48,776
75 Centile	45,962	47,567	49,507	51,952	55,018	57,405	59,657	61,440
Probability of Firm Switch^2^	0.11	0.10	0.08	0.09	0.08	0.07	0.06	0.06
Probability of Industry Switch^3^	0.09	0.08	0.07	0.08	0.08	0.07	0.06	0.06
Probability of Occupation Switch^4^	0.15	0.12	0.11	0.12	0.14	0.10	0.08	0.07

All experiences are averages measured in years; ^1^Net annual income after tax in Euros; ^2^Probability of firm switch within the next year; ^3^Probability of industry switch within the next year; ^4^Probability of occupation switch within the next year;

### Graphical analysis

Figures 1, 2 and 3 capture the nonlinear relationships between firm, industry, and sales occupation experience and percentage increase in compensation. The original dependent variable is log of net total compensation. Therefore, the changes in the coefficients should be interpreted as the percentage change in the dependent variable. The diminishing returns for all three types of experiences indicate a point after which adding to the given experience yields no further returns.

Figure 1 plots the returns to firm experience. Interestingly, for both groups, returns to firm experience plateau after about three years (2.9 and 3.4 years for salespeople and sales managers, respectively), at 3% for sales managers and 1.1% for salespeople, beyond which point the cumulative returns stay relatively stable for sales managers at 3% but decrease to 1.9% (down 0.2%) for salespeople. Notably, a decreasing marginal return (from 1.5% to 1.3%) does not mean that the salesperson’s salary will decrease. The decreasing marginal return is relative to the salesperson’s opportunity cost, meaning that when salespeople stay in the firm beyond the third year, they forego 0.2% of the additional income they would have gained by switching to another firm. Similarly, Fig. 2 shows that returns to industry experience for sales managers are steeper, and they peak after 3.6 years at 1.9% for sales managers and after 3.1 years at 0.6% for salespeople. Perhaps the most interesting finding is that returns to occupation experience are much steeper and diminish more slowly than returns to firm and industry experience for both groups. However, there is a substantial difference between sales managers and salespeople. The returns to occupation experience peak after 6.4 years at 13.8% for sales managers, whereas for salespeople, it peaks much earlier, after 3.4 years and at 3.25%. After this point, the marginal return decreases, albeit at a much slower rate than firm experience. This is in line with the hypothesized (H2a and H2b) importance of occupation experience.

**Fig. 4:**
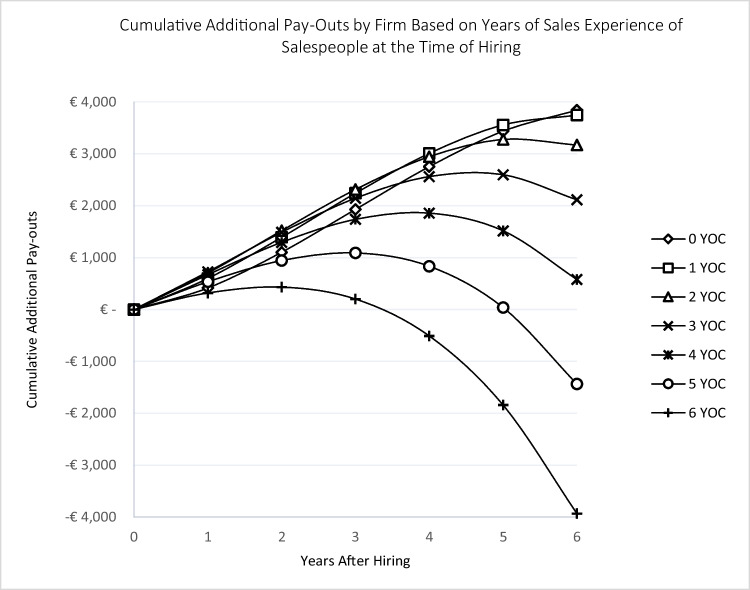
Salespeople occupation experience and cumulative pay-outs by firm Note: YOC: Years of Occupation Experience; For example, a salesperson on the curve marked by square had one year of sales experience when joining the firm. The additional pay-out by the firm after six years, based on all types of accumulated experiences, is about 3,745 euros.

**Fig. 5:**
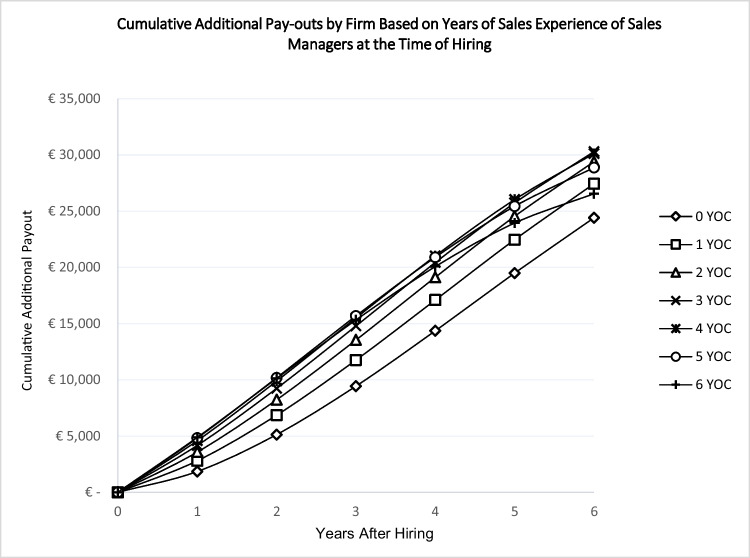
Sales managers occupation experience and cumulative pay-outs by firm Note: YOC: Years of Occupation Experience. For example, a sales manager on the curve marked by square had one year of sales experience when joining the firm. The additional pay-out of the firm after six years, based on all types of accumulated experiences, is about 27,444 euros.

In a further analysis, we run simulations using the marginal effects of the models presented in Table 4, depicted in Figs. 4 and 5, which represent the *additional* cumulative pay-outs of firms to salespeople and sales managers, respectively, by years after hiring. As can be seen, there is a difference between the least and most experienced hires in terms of additional pay-out as time passes. The gap is around 8,000 Euros for salespeople after six years in the firm. Interestingly, this gap between the least and most experienced sales managers does not widen as sharply as for salespeople: the difference is about 5,700 Euros.

### Comparative analyses

To assess confidence in the validity of the results, we compare sales to accounting and human resource jobs, shedding further light on the underlying mechanisms. Indeed, both professions share several common characteristics with sales jobs.

First, salespeople have long been portrayed as spanning the boundary between the firm and its customers: developing, maintaining, and expanding customer relationships (Bradford et al., 2010). Much like salespeople, accountants represent the face of their firm as they uncover their customers’ issues, understand the reporting implications, communicate, and explain their firm’s concerns to various client personnel (Jelinek & Boyle, 2022). Accountants are also portrayed as boundary spanners because they collaborate with other professionals both within their firm and at client organizations (Dekker, 2016; Jelinek & Boyle, 2022). Similarly, HR professional responsibilities include a strategic role representing their organization as they keep track of talent need evolution, customer, and investor expectations, and changing business conditions (Ulrich et al., 2007).

Second, in B2B field sales, a broad spectrum of sales jobs ranges from those that demand little know-how or problem-solving when working with transactional customers to complex jobs involving consultative relationship management and internal cross-functional interactions with strategic accounts (Rouziès et al., 2009). Much like sales jobs, accounting jobs are very diverse, from jobs that simply focus on recording financial transactions to public accounting jobs demanding technical knowledge of reporting implications of client issues and the ability to explain a firm’s concerns to client personnel (Jelinek & Boyle, 2022). In the same vein, HR roles span a wide array of administrative, people-related, organizational, and strategic responsibilities (Galang & Osman, 2016).

Third, the sales profession is well known for its high turnover rate (Boles et al., 2012; Northon et al., 2016). Given that much of this turnover is voluntary, barriers to the mobility of sales professionals are likely to be low. Accounting and HR are also plagued with high exit rates (Robson et al., 1996). Thus, accounting and HR are prototypical examples of professional services requiring expertise and skills that are transferable across firms (Pennings et al., 1998; Teece, 2003; Von Nordenflycht, 2010).

In order to compare sales to accounting and HR jobs, we determined the yearly compensation of 4,562 accounting managers over 19,812 accounting manager-year-firm observations, and 9,861 HR managers over 41,176 HR managers-year-firm observations, then ran separate and pooled analyses comparing the results with those of sales managers. Because accounting and HR jobs are generally less *firm*-specific than sales jobs (given the importance of internal networks for sales to be generated), we expect that returns to experience will be lower for accounting and HR than for sales managers. Given that accounting jobs are associated with high *occupation* specificity, we would further expect that accounting managers obtain, as do sales managers, strong levels of return to occupation experience. However, as HR is not considered an established profession (Hallier and Summers, 2011), we expect that HR managers obtain lower levels of return to *occupation* experience compared to sales managers. Note that because we have no theoretical basis for predicting accounting or HR managers’ payoff for *industry* experience, we limit the comparisons to firm and occupation experience. We include this empirical test in Table 7.

**Table 7:** Two-stage least square regressions for sales, accounting, and HR managers including combined instrumental variables

	Sales Managers	Accounting Managers	HR Managers	Sales and Accounting	Sales and Accounting	Sales and Accounting	Sales and HR	Sales and HR	Sales and HR
Variable	Model 1	Model 2	Model 3	Model 4	Model 5	Model 6	Model 7	Model 8	Model 9
Industry Experience	0.011*** (0.001)	0.008*** (0.002)	0.024*** (0.003)	0.012*** (0.001)	0.010*** (0.001)	0.010*** (0.001)	0.022*** (0.003)	0.020*** (0.003)	0.020*** (0.003)
Industry Experience^2^	-0.002*** (2.0E-4)	-0.001*** (3.1E-4)	-0.001*** (1.6E-4)	-0.002*** (2.0E-4)	-0.002*** (2.0E-4)	-0.002*** (2.0E-4)	-0.002*** (1.9E-4)	-0.002*** (1.8E-4)	-0.002*** (1.8E-4)
Firm Experience	0.018*** (0.001)	0.001 (0.002)	-0.006 (0.003)	0.003* (0.001)	0.005*** (0.001)	0.002* (0.001)	0.002* (0.001)	0.003* (0.001)	0.002* (0.001)
Firm. Experience^2^	-0.004*** (2.3E-4)	-0.001 (3.8E-4)	-1.0E-4 (1.7E-4)	-0.001*** (2.4E-4)	-0.001*** (2.5E-4)	-0.001** (2.4E-4)	-1.0E-4 (2.4E-4)	-1.0E-4 (2.4E-4)	-1.0E-4 (2.4E-4)
Occupation Experience	0.037*** (0.001)	0.039*** (0.002)	0.023*** (0.003)	0.034*** (0.0013)	0.034*** (0.001)	0.035*** (0.001)	0.027*** (0.002)	0.027*** (0.002)	0.028** (0.002)
Occupation Experience^2^	-0.003*** (3.8E-4)	-0.004*** (0.001)	-0.001*** (2.0E-4)	-0.003*** (4.2E-4)	-0.003*** (4.2E-4)	-0.003*** (4.2E-4)	-0.002*** (2.6E-4)	-0.002*** (2.6E-4)	-0.002*** (2.1E-4)
S.M.^1^ × Industry Experience				0.010*** (0.001)			-0.012*** (0.001)		
S.M. x Industry Experience^2^				-4.4E-4 (3.1E-4)			0.001 (0.001)		
S.M. x Firm Experience					0.011*** (0.001)			0.014*** (0.001)	
S.M. x Firm. Experience^2^					-5.4E-4 (3.4E-4)			-0.001** (4.0E-4)	
S.M. x Occupation Experience						4.7E-4 (0.002)			0.015*** (0.001)
S.M. x Occupation Experience^2^						0.001 (0.001)			-0.001*** (2.5E-4)
S.M				-0.064*** (0.008)	-0.068*** (0.008)	-0.062*** (0.009)	-0.052*** (0.007)	-0.055*** (0.008)	-0.051*** (0.007)
All Control Variables	Yes	Yes	Yes	Yes	Yes	Yes	Yes	Yes	Yes
Number of Observation	21,994	19,182	45,396	41,176	41,176	41,176	67,390	67,390	67,390
Number of Employees	5,299	4,562	11,081	9,861	9,861	9,861	16,380	16,380	16,380
Kleibergen-Paap Wald rk F	748	745	1,619	1,244	1,168	808	1,769	1,769	1,769
R^2^	0.655	0.591	0.611	0.619	0.617	0.610	0.628	0.628	0.628

^*^ *p* < 0.05, ** *p* < 0.01, *** *p* < 0.001; The dependent variable is the log total net compensation; Heteroskedasticity and autocorrelation robust (HAC) estimation standard errors adjusted for clustering at the firm level are reported in parentheses. ^1^ S.M. stands for Sales Managers

Broadly, our findings correspond to our expectations. In particular, Model 2 [Model 3] confirms that firms do not significantly value experience within the same *firm* for accounting and [HR] managers. Further, Model 5 [Model 8] shows that although sales managers are generally paid less (β = -0.068, *p* < 0.001) [β = -0.055, *p* < 0.001] than accounting [HR] managers, their return to *firm* experience is significantly higher than it is for their accounting [HR] counterparts (β = 0.011, *p* < 0.001) [β = 0.014, *p* < 0.001]. In Model 2, we also found evidence that career trajectory (i.e., *occupation* experience) is important for accounting managers. In fact, each additional year of *occupation* experience brings them a 3.9% annual pay increase (*p* < 0.001). Interestingly, sales managers also get a high return to *occupation* experience (3.7% annual pay increase for each additional year of *occupation* experience, *p* < 0.001), as shown in Model 1. Thus, there is no significant difference between accounting and sales managers’ returns to *occupation* experience, as seen in Model 6. As expected, however, HR managers’ return to *occupation* experience is significantly lower than that of sales managers (β = 0.015, *p* < 0.001), as shown in Model 9.

## Discussion

### Contributions

The contributions of this paper are four-fold.

#### Revisiting salesforce compensation

First, our study revisits the issue of salesforce compensation. To the best of our knowledge, compensation research in marketing rarely considers the past experience of salespeople and sales managers when deciding how much they should earn. In fact, prior research in marketing mostly emphasizes the motivational role of compensation for salespeople. With this research gap in mind, we examine another element of the compensation equation and investigate how firms value sales career paths. Due to the ubiquity of sales, sales employees are found in a diverse range of jobs, organizations, and industries. This diversity allows a rare examination of how firm, industry, and sales experience drive compensation.

#### Using an instrumental-variable method

Second, our instrumental-variable method, seldom used in sales research, permits us to go beyond simple individual fixed-effect methods to control for individual performance heterogeneity while also accounting for heterogeneity in firms, industries, and occupations and their match to the abilities and motivation of heterogeneous individuals. Given the exceptional breadth and depth of information available in our data (sales employee careers over 22 years across firms, industries, and occupations), we capture patterns in pay levels at the individual career history level of salespersons and sales managers and show how sales career paths affect their compensation, a topic never investigated in sales research to the best of our knowledge.

#### Assessing how firms value sales career paths

Third, we suggest that the impact of career paths on compensation at the sales-management level does not replicate the impact documented at the salespeople level. As shown in Table 8, our findings that sales managers’ occupation, firm, and industry experience are more highly valued than that of salespeople are consistent with the observed multiplier effect of sales managers (Elling et al., 2002), which justifies paying them at increasing rates as firm, industry, or sales occupation experience accumulates (Cappelli & Cascio, 1991; Ortin-Angel & Salas-Fumas, 2007; Rouziès et al., 2009). In addition, our results show an inverted U-shaped relationship between experience and compensation that is in keeping with the hypothesized combination of accrued skills and knowledge, “technological evolution”, and “achievement weariness” mechanisms (Cron & Slocum, 1986; Homburg et al., 2010; Kanfer & Ackerman, 2004). In other words, our analyses suggest that firms should adapt their compensation schemes to account for the declining skills and knowledge obsolescence of sales employees. Additionally, we find direct evidence that sales-specific experience is more financially rewarding than firm-specific or industry-specific experience for both salespeople and sales managers *during their career* (accounting for non-sales managerial experience). These effects align with our conception of the *"sales identity"* that drives a compensation premium because firms typically view *sales* roles as strategic and value a focused *sales* career history. These results also reveal that firms view the development of sales competencies (1) as a long-term investment, (2) as transferable across firms and industries (i.e., more general than *industry*- or *firm*-specific skills), thereby more likely to be valued by external employers, and (3) as informing their compensation decisions (i.e., *more than firm* or *industry* experience). Interestingly, this rank ordering of sales employees’ expertise (i.e., sales occupation compared to industry and firm experiences) reflects the dominant beliefs regarding necessary competencies.

**Table 8:** Summary results on returns to experience for sales employees

Characteristics		Salespeople	Sales Managers
Firm Experience	Length of Experience to Reach Plateau	2.9 years	3.4 years
	Cumulative % of Compensation Added as a Result of Experience at Plateau	1.1%	3%
Industry Experience	Length of Experience to Reach Plateau	3.1 years	3.6 years
	Cumulative % of Compensation Added as a Result of Experience at Plateau	.6%	1.9%
Sales Experience	Length of Experience to Reach Plateau	3.4 years	6.4 years
	Cumulative % of Compensation Added as a Result of Experience at Plateau	3.25%	13.8%

#### Uncovering career paths in sales organizations

Fourth, we establish the importance of career paths for designing compensation plans. Hence, another implication of our paper is that firms may wish to develop “internal pipelines” (Brymer et al., 2019) to adapt to the challenges of asymmetric information and talent management, as presented in Table 9. Indeed, we find that company leaders choose sales managers mostly from within their firm (about 3 out of 4). Given that it is (1) less costly to ascertain internal than external candidates’ skills and (2) easier to develop talent internally than access external talent, it is not surprising that firms staff their sales-management positions with their own employees. Notably, this pattern mirrors a policy of preferentially recruiting sales managers from other managerial functions (about 2 out of 3) and with higher remuneration levels (€42,881 without vs. €34,303 with sales experience). This is consistent with the fact that managers from other functions—who typically have accumulated between 2 and 2.5 years of non-sales managerial experience—may know less about the sales-management position than salespeople from the same firm, and thereby demand more pay to compensate for their mobility risk. Indeed, costs of misplacements are likely to be important: newly promoted sales managers without sales experience are more likely to switch occupations (30%) the year after becoming sales managers compared to managers with sales experience (15%) within one year after becoming sales managers. Moreover, newly promoted sales managers without sales experience have less industry (3.55 years vs. 6.20 years, respectively), firm (3.16 years vs. 5.23 years), and work (9.85 vs. 11.38 years) experience than their counterparts with sales experience. Taken together, these observations show how the development of internal pipelines highlights the paradox of sales. Thus, for staff *sales-management jobs*, firms prefer previous non-sales managerial experience, thereby discounting sales experience. Consequently, our study implies that firms generally value management skills more than sales skills when choosing sales managers.^8^ As seen in Web Appendix F, the additional return that each year of sales experience adds to a newly promoted sales manager’s compensation will not make up for the gap between management and sales skills value. Interestingly, after two years in sales-management jobs, sales managers with prior non-sales managerial experience are more likely to stay in sales-management jobs and move up the ranks more rapidly than their counterparts with sales experience. During their career, sales managers (like salespeople) earn a premium for staying in sales jobs. All in all, we uncover two distinct career paths in sales organizations: (1) the salesperson’s career path rewarding a “sales identity” (i.e., rewarding sales experience better than firm or industry experience) but less likely to lead to sales-management jobs; and (2) another path, a paradox, providing promotional prospects in sales management but generally starting from another managerial role in the same firm.

**Table 9:** Summary results on sales management careers, work experience and compensation

**Characteristics**		Generally More Successful ^1^ Sales Management Careers	Generally Less Successful Sales Management Careers
Length of Experience at Time of Promotion in Sales Management	Firm Experience	**-**	** + **
	Industry Experience	**-**	** + **
	Sales Experience	**-**	** + **
	Work Experience	**-**	** + **
Managerial Experience other than Sales Management Experience		** + **	**-**
Compensation Level		** + **	**-**
External Mobility	At Year of Promotion in Sales Management	** + **	**-**
	After 2 Years in Sales Management	**-**	** + **

Notes:^**1**^ Success is defined in terms of financial rewards and speed of upward mobility; +  = indicates that sales managers (i.e., either more or less successful depending on column) have more of a given characteristic (i.e., row);—= indicates that sales managers (i.e., either more or less successful depending on column) have less of a given characteristics (i.e., row)

### Future research and limitations

An important question related to our topic of interest was raised by Leung (2014). Is there an order of accumulated experiences that is more rewarded than others? Some researchers (e.g., Homburg et al., 2008) recommend career paths featuring sales and marketing experience. Therefore, it is worthwhile for future research to know whether organizations promote career paths focused first on sales and then on marketing, more than purely sales-focused experience.

Other work could also explore the sales career paths leading to the upper echelons of firms. For example, further research could investigate how the sales experience of higher-level executives affects firm strategy and performance.

Although our instrument does not completely account for changes in individual characteristics and motivation over time, it goes a long way toward estimating the effect of various types of experiences on sales personnel earnings. Using this instrumental-variable method, future researchers could investigate other noteworthy aspects of careers in sales. For example, we know little about whether firms benefit by rehiring former salespeople or sales managers. It would also be interesting to know the performance difference between the two career paths presented in Table 9.

All in all, such research studies will benefit from more fine-grained categorizations of jobs and outcome variables than our data provide.

### Managerial and organizational implications

Our research offers several suggestions for decision-makers. In particular, our findings have important implications for firms transitioning managers from other managerial functions to sales management. As noted earlier, these transferred managers are better paid than their counterparts with sales experience. Sales leaders are advised to pair this promotion pathway with specific training to help employees succeed and induce them to stay with the firm. We found that internally sourced sales managers without sales experience are more likely to switch firm, industry, and occupation than their colleagues with sales experience.

Taken together, these findings also offer useful insights for sales managers and salespeople, who may wonder how to manage their own careers and must decide whether to remain in the same industry, in the same firm, or even stay in the same occupation. Sales managers and salespeople probably want to know how career choices affect their lifetime earnings. Clearly, both will be better off financially if they are open to moving to other firms or other industries about three years after joining a firm. Interestingly, despite the rewards associated with their inter-organizational or cross-industry mobility, as salespeople become more established in the occupation, their mobility across firms or industries declines (see Table 6). We surmise that salespeople stay focused on one firm and industry because of personal choice, job satisfaction, career stage, loyalty, or inertia. Alternatively, fear of getting out of a comfort zone, coupled with the range of expertise and networks required for salespeople to remain effective, may also impose job stickiness.^9^ It is possible that a successful, accomplished salesperson may want additional non-monetary rewards, less competitive environments (Miao et al., 2009), more control over their time, or better work/life balance (Cron et al., 1988). We also suspect that salespeople in this career stage accept their earnings potential. As a result, maintaining their income at satisfactory levels without performing challenging tasks may be more of a priority for them (Miao et al., 2009).

Sales managers will generally do better if they stay at least 6.4 years in their position before switching occupation, whereas salespeople will be better off if they stay about 3.4 years in their occupation. However, salespeople generally avoid switching occupations, as shown in Table 6, probably for the reasons presented above. In light of today’s context of the Great Resignation, firms can expect that some salespeople will leave. In this case, firms are better off hiring salespeople with more sales experience, as can be seen in Fig. 4. As Fig. 5 shows, there is a much slower decline in the additional pay-outs based on sales experience to sales managers than to salespeople at the time of hiring. Again, given today’s context, firms can expect that some sales managers will leave. In this case, firms are better off hiring sales managers with more sales experience. Given that there are far fewer sales managers than salespeople in a firm, the savings related to rehiring sales managers with sales experience are more limited. However, given sales managers’ multiplier effect, rehiring a sales manager has a potentially greater financial impact on the firm than rehiring salespeople. In a nutshell, these compensation savings comparisons should be weighed against the financial impact of rehiring a new employee in a sales role.

Perhaps the most interesting insight is the “sales paradox” we uncover. Clearly, sales management is not a continuation of a salesperson job. In fact, the career strategies of sales managers and salespeople are not the same. First, it is best to choose other managerial occupations than sales if one wants to access sales management, as firms are more likely to use lateral transfers at the managerial level than promoting from the sales function. Second, even the rewards associated with the length of stay in the same specialization are not similar for the two groups. Consider the following scenario to illustrate the importance of our findings. A sales manager with a five-year career in sales in the same firm will increase his/her earnings by 9% more than a sales manager with little experience in sales (for example, one year in sales and four years of other non-managerial jobs) but in the same firm. The same strategy will not pay off as much for a salesperson, who would earn only 2.5% more than his/her counterpart with little sales experience.Also, everything else being equal, a sales manager would increase his/her income by 1.9% by staying in the same industry for 3.5 years, while there would be a very little payoff, 0.6%, for a salesperson who does the same.

Taken together, our results suggest that decision-makers take career trajectories into account when designing their compensation plans, an insight that deserves further scrutiny, given the critical importance of salespeople and sales managers to a firm's success.

## Supplementary Information

Below is the link to the electronic supplementary material.

Supplementary file1 (DOCX 110 KB)

## Footnotes

1 We thank the anonymous reviewers for pointing out this literature strand.

2 We thank an anonymous reviewer for that suggestion.

3 A separate analysis (available upon request) is conducted on a sample of salespeople who go on to become sales managers, where sales jobs—whether sales manager or salesperson—are treated as one occupation. The analysis yields results consistent with the main findings of the paper. We thank an anonymous reviewer for this clarification.

4 Other sources of endogeneity, where firm-to-firm mobility is related to time-varying innovations in the wage equation, cannot be addressed with this type of empirical strategy. Structural estimation (see Rutz and Watson 2019) would help alleviate such problems but might still require exclusion restrictions to cleanly identify the various returns to experience. To the best of our knowledge, the literature does not offer a convincing strategy to estimate these returns.

5 The constructed variable instrument using Altonji and Shakotko's (1987) method to instrument firm experience is designed to capture the time-invariant (mis)match effects between an individual and a firm. This error can spill over to the estimation of other correlated variables, i.e., industry and occupation experience in an additive model such as the one presented in this paper. By subtracting the mean of firm experience from all the observations of an individual in a firm (a job spell), the match effect is canceled out of the firm experience, thereby differencing out this error term. Simultaneously, similar instruments are constructed to capture the match effect between individuals and their occupations. Finally, a third instrument is generated to capture the match effect between individuals and industries. The spillover effect is eliminated because all these instruments, together with the heteroskedasticity-based instruments (HBIVs), enter the equation as a vector at the same time.

6 We thank a referee for suggesting this strategy.

7 We thank an anonymous reviewer for suggesting that we account for non-sales managerial experience in this model.

8 We thank an anonymous reviewer for suggesting this point.

9 We thank an anonymous reviewer for suggesting this point.
