# Supplementary material for: How do firms value sales career paths?
Source: J Acad Mark Sci. 2023 Jun 17;52(3):762–88. doi: 10.1007/s11747-023-00952-4 (PMC11636709; doi:10.1007/s11747-023-00952-4)
Supplement: Supplementary file 1 — Supplementary file1 (DOCX 110 KB) [file 11747_2023_952_MOESM1_ESM.docx]

Web Appendix

| Web Appendix A | Underlying Mechanisms for Hypothesized Relationships |
| --- | --- |
| Web Appendix B | Flow Chart of Sample Generation and Experience Measure Construction |
| Web Appendix C | List of Variables |
| Web Appendix D | Mobility Statistics |
| Web Appendix E | Summary Statistics on Salespeople Categorized by Sales Occupation Experience |
| Web Appendix F | Summary Statistics on Newly Promoted Sales Managers Categorized by Sales Occupation Experience |

**A.1: The Underlying Mechanism of the Inverted U-Shaped Effect (H1)**

KSAs Benefits

Upskilling Costs

Compensation

Firm/Industry/Sales Occupation Exp.

Firm/Industry/Sales Occupation Exp.

Firm/Industry/Sales Occupation Exp.

**A.2: The Underlying Mechanism of the Moderated Inverted U-Shaped for Heterogeneity of Work Experience (H2)**

KSAs Benefits

Upskilling Costs

Compensation

Experience

Experience

Experience

Sales Occupation Experience of Salespeople

Firm and Industry Experience of Salespeople

**A.3: The Underlying Mechanism of the Moderated Inverted U-Shaped for Job Position (H3)**

KSAs Benefits

Upskilling Costs

Compensation

Sales Experience

Sales Experience

Sales Experience

Sales Managers

Salespeople

Web Appendix A Underlying Mechanisms for Hypothesized Relationships

Web Appendix B Flow Chart of Sample Generation and Experience Measure Construction

Note: The initial dataset contains every observation of every worker in the economy who entered the labor market between 1994 and 2015. For the first sample, individuals were included if they had worked at least 90 days in a full-time job with one of the 27 salespeople or 8 sales manager occupation codes and in one of the 20 manufacturing industry codes. This yields 5,731 sales managers and 23,918 salespeople over 58,334 and 193,017 observations, respectively. Then the experience variables and their respective instruments are generated as follows.

For each observation of each individual in a given year, the *previous observation* of that individual is screened to generate (1) industry experience by adding all the previous periods worked in *two-digit industry codes* of the focal observation, (2) firm experience by adding all the previous periods worked in the *firm (coded under the SIREN scheme by the French administration)* of the focal observation, (3) salesperson and sales managers occupation experience by adding all the previous periods worked in the *salesperson or sales manager occupation codes* of the focal observation.

Finally, to have a meaningful measure of experience and career, only the sales management and salesperson observations of individuals with more than two years of total work experience and more than one year of sales occupation experience are included in the final sample for analysis. Please note that we kept people with only the sales occupation experience of individuals as the occupation experience measure corresponded to the occupation experience of interest. In theory, we could add a measure of non-sales occupations, but that measure would be a linear combination of the sales occupation measure and total work experience, which is already included in the model.

| Web Appendix D.1 Mobility Statistics of Salespeople Within the Next Year Starting with Firm Switch | | | | | | | | |
| --- | --- | --- | --- | --- | --- | --- | --- | --- |
| Firm Switch | Yes  8,744  (11%) | | | | No  69,479  (89%) | | | |
| Industry Switch | Yes  5,255  (60%) | | No  3,489  (40%) | | Yes  0  (0%) | | No  69,479  (100%) | |
| Occupation Switch | Yes  3,333 (63%) | No  1,921 (37%) | Yes  673 (19%) | No  2,816 (81%) | Yes  0  (0%) | No  0  (0%) | Yes  5,763  (8%) | No  63,716 (92%) |

| Web Appendix D.2 Mobility Statistics of Salespeople Within the Next Year Starting with Occupation Switch | | | | | | | | |
| --- | --- | --- | --- | --- | --- | --- | --- | --- |
| Occupation Switch | Yes  9,770  (12%) | | | | No  68,453  (88%) | | | |
| Firm  Switch | Yes  4,007  (41%) | | No  5,763  (59%) | | Yes  4,737  (55%) | | No  63,716  (45%) | |
| Industry Switch | Yes  3,333 (83%) | No  673 (17%) | Yes  0 (0%) | No  5,763 (100%) | Yes  1,921 (41%) | No  2,816 (59%) | Yes  0 (0%) | No  63,716 (100%) |

| Web Appendix D.3 Mobility Statistics of Sales Managers Within the Next Year Starting with Firm Switch | | | | | | | | |
| --- | --- | --- | --- | --- | --- | --- | --- | --- |
| Firm  Switch | Yes  3,209  (9%) | | | | No  32,345  (91%) | | | |
| Industry Switch | Yes  2,053  (64%) | | No  1,156  (36%) | | Yes  0  (0%) | | No  32,345  (100%) | |
| Occupation Switch | Yes  1,082 (53%) | No  971 (47%) | Yes  346 (30%) | No  810 (70%) | Yes  0 (0%) | No  0 (0%) | Yes  3,328 (10%) | No  29,017 (90%) |

| Web Appendix D.4 Mobility Statistics of Sales Managers Within the Next Year Starting with Occupation Switch | | | | | | | | |
| --- | --- | --- | --- | --- | --- | --- | --- | --- |
| Occupation Switch | Yes  4,756  (13%) | | | | No  30,798  (87%) | | | |
| Firm  Switch | Yes  1,428  (30%) | | No  3,328  (70%) | | Yes  1,781  (6%) | | No  29,017  (94%) | |
| Industry Switch | Yes  1,082 (76%) | No  346 (24%) | Yes  0 (0%) | No  3,328 (100%) | Yes  971 (55%) | No  810 (45%) | Yes  0 (0%) | No  29,017 (100%) |

| Web Appendix E Summary Statistics on Salespeople Categorized by Sales Occupation Experience | | | | | | | | |
| --- | --- | --- | --- | --- | --- | --- | --- | --- |
|  | Number of Years of Sales Experience | | | | | | | |
|  | 2 Years | 3 Years | 4 Years | 5 Years | 6 Years | 7 Years | 8 Years | 9 Years |
| Number of Observations | 13,196 | 10,908 | 9,255 | 7,780 | 6,513 | 5,465 | 4,482 | 3,673 |
| Probability of Becoming SM^1^ | 0.22 | 0.22 | 0.21 | 0.21 | 0.20 | 0.18 | 0.17 | 0.16 |
| Industry Experience | 3.12 | 4.02 | 4.94 | 5.88 | 6.83 | 7.76 | 8.70 | 9.67 |
| 25 centile | 2.00 | 3.00 | 3.99 | 4.99 | 6.00 | 7.00 | 8.00 | 9.00 |
| 50 centile | 2.83 | 3.88 | 4.91 | 5.92 | 7.00 | 7.90 | 9.00 | 10.00 |
| 75 centile | 3.08 | 4.16 | 5.21 | 6.25 | 7.30 | 8.40 | 9.50 | 10.80 |
| Company Experience | 2.80 | 3.59 | 4.40 | 5.22 | 6.00 | 6.80 | 7.65 | 8.48 |
| 25 centile | 1.78 | 2.61 | 3.41 | 4.00 | 4.70 | 5.20 | 6.00 | 6.90 |
| 50 centile | 2.33 | 3.32 | 4.31 | 5.30 | 6.30 | 7.00 | 8.10 | 9.20 |
| 75 centile | 3.00 | 4.00 | 5.00 | 6.00 | 7.00 | 8.00 | 9.00 | 10.00 |
| Sales Occupation Experience | 1.98 | 2.98 | 3.98 | 4.98 | 5.98 | 6.98 | 7.98 | 8.98 |
| Total Work Experience | 7.65 | 8.60 | 9.54 | 10.47 | 11.43 | 12.43 | 13.26 | 14.14 |
| 25 centile | 3.00 | 4.00 | 5.00 | 6.50 | 7.00 | 9.00 | 10.00 | 11.00 |
| 50 centile | 6.00 | 7.00 | 8.00 | 9.50 | 10.00 | 11.00 | 12.00 | 13.00 |
| 75 centile | 9.00 | 10.00 | 11.00 | 12.50 | 13.00 | 14.00 | 15.00 | 15.00 |
| Net Annual Compensation^2^ | 22,980 | 23,735 | 24,644 | 25,509 | 26,215 | 26,941 | 27,618 | 28,399 |
| 25 centile | 16,635 | 17,963 | 19,019 | 20,020 | 20,930 | 21,658 | 22,204 | 23,114 |
| 50 centile | 20,748 | 22,040 | 23,169 | 24,206 | 24,934 | 25,662 | 26,572 | 27,300 |
| 75 centile | 26,026 | 26,954 | 28,010 | 28,756 | 29,484 | 30,394 | 31,122 | 32,032 |
| Probability of Firm Switch^3^ | 0.14 | 0.11 | 0.1 | 0.08 | 0.06 | 0.06 | 0.06 | 0.05 |
| Probability of Industry Switch^4^ | 0.09 | 0.07 | 0.06 | 0.06 | 0.05 | 0.05 | 0.04 | 0.04 |
| Probability of Occupation Switch^5^ | 0.14 | 0.11 | 0.1 | 0.09 | 0.11 | 0.08 | 0.08 | 0.08 |
| Time to Becoming Sales Manager^6^ | 5.88 | 5.53 | 5.14 | 4.77 | 4.44 | 4.11 | 3.73 | 3.38 |
| 25 centile | 3.00 | 3.00 | 2.00 | 2.00 | 2.00 | 2.00 | 2.00 | 2.00 |
| 50 centile | 5.00 | 5.00 | 5.00 | 4.00 | 4.00 | 4.00 | 3.00 | 3.00 |
| 75 centile | 9.00 | 8.00 | 8.00 | 7.00 | 7.00 | 6.00 | 5.00 | 5.00 |
| All experiences are averages measured in years; ^1^SM: Sales Manager; ^2^ Net annual income after tax; ^3^Probability of firm switch within the next year; ^4^Probability of industry switch within the next year; ^5^Probability of occupation switch within the next year; ^6^Average number of years before the salesperson becomes sales manager. | | | | | | | | |

| Web Appendix F Summary Statistics on Newly Promoted Sales Managers Categorized by Sales Occupation Experience | | | | | | | | | | | | |  |
| --- | --- | --- | --- | --- | --- | --- | --- | --- | --- | --- | --- | --- | --- |
|  | Newly Promoted Sales Managers With: | | | | | | | | | | | |  |
|  |  | |  | |  | | | | | | | |  |
| Variable | No Managerial  Experience | Managerial  Experience | No Salesperson  Experience | Salesperson Experience | 2 Years Salesp. Exp. | 3 Years Salesp. Exp. | 4 Years Salesp. Exp. | 5 Years Salesp. Exp. | 6 Years Salesp. Exp. | 7 Years Salesp. Exp. | 8 Years Salesp. Exp. | 9 Years Salesp. Exp. | |
| Number of Observations | 2,651 | 2,934 | 3,744 | 1,841 | 368 | 277 | 222 | 176 | 162 | 176 | 98 | 92 | |
| Industry Experience | 4.34 | 4.50 | 3.55 | 6.20 | 3.78 | 4.40 | 4.65 | 5.77 | 6.47 | 7.47 | 8.37 | 9.53 | |
| 25 centile | 1.00 | 2.00 | 1.00 | 2.81 | 1.57 | 2.66 | 3.00 | 4.49 | 5.30 | 6.72 | 7.87 | 8.91 | |
| 50 centile | 3.66 | 3.38 | 2.90 | 5.58 | 3.00 | 3.97 | 4.54 | 6.00 | 6.99 | 8.00 | 8.99 | 10.00 | |
| 75 centile | 7.00 | 7.00 | 5.00 | 9.00 | 4.83 | 5.29 | 5.33 | 7.00 | 7.89 | 9.00 | 10.00 | 11.00 | |
| Firm Experience | 3.75 | 3.92 | 3.16 | 5.23 | 3.24 | 3.77 | 3.85 | 4.85 | 5.02 | 6.43 | 7.23 | 8.18 | |
| 25 centile | 1.00 | 1.40 | 1.00 | 1.00 | 1.00 | 1.00 | 1.00 | 1.69 | 1.00 | 2.67 | 3.67 | 5.00 | |
| 50 centile | 2.96 | 3.00 | 2.00 | 4.00 | 2.33 | 3.00 | 4.00 | 5.00 | 5.75 | 7.75 | 8.32 | 9.15 | |
| 75 centile | 6.00 | 6.00 | 4.25 | 8.00 | 3.94 | 4.77 | 5.00 | 6.28 | 7.00 | 8.00 | 9.92 | 11.00 | |
| Total Work Experience | 9.99 | 10.94 | 9.85 | 11.38 | 9.37 | 10.07 | 9.92 | 10.96 | 11.31 | 12.12 | 12.87 | 14.19 | |
| 25 centile | 5.00 | 5.00 | 4.00 | 8.00 | 5.00 | 6.00 | 6.00 | 7.00 | 7.00 | 8.00 | 10.00 | 11.00 | |
| 50 centile | 8.00 | 9.00 | 8.00 | 11.00 | 8.00 | 9.00 | 9.00 | 10.00 | 10.00 | 10.00 | 12.00 | 13.00 | |
| 75 centile | 12.00 | 13.00 | 11.00 | 14.00 | 12.00 | 12.00 | 1200 | 13.00 | 13.00 | 14.00 | 14.00 | 16.00 | |
| Sales Occupation Exp. at Promotion | 2.66 | 0.91 | 0.00 | 5.39 | 1.96 | 2.98 | 3.99 | 4.98 | 5.98 | 6.98 | 8.00 | 8.98 | |
| Net Annual Compensation^1^ | 33,051 | 46,380 | 42,881 | 34,303 | 33,532 | 34,162 | 32,810 | 34,058 | 34,856 | 34,968 | 35,068 | 34,356 | |
| 25 centile | 25,553 | 32,787 | 28,921 | 27,301 | 25,784 | 26,208 | 25,773 | 27,142 | 27,897 | 29,060 | 28,775 | 29,059 | |
| 50 centile | 30,503 | 39,967 | 37,417 | 32,190 | 31,177 | 31,567 | 30,438 | 31,461 | 32,170 | 33,235 | 33,181 | 32,832 | |
| 75 centile | 37,401 | 50,196 | 47,625 | 38,707 | 38,180 | 38,476 | 37,245 | 38,408 | 37,481 | 39,588 | 40,543 | 37,861 | |
| Coming from Different Firm^2^ | 0.28 | 0.25 | 0.27 | 0.26 | 0.32 | 0.27 | 0.32 | 0.25 | 0.27 | 0.20 | 0.16 | 0.15 | |
| Coming from Different Industry^3^ | 0.20 | 0.20 | 0.21 | 0.17 | 0.23 | 0.18 | 0.22 | 0.14 | 0.15 | 0.15 | 0.11 | 0.09 | |
| Coming from Other Managerial Jobs^4^ | 0.00 | 0.87 | 0.65 | 0.14 | 0.17 | 0.16 | 0.14 | 0.12 | 0.12 | 0.17 | 0.14 | 0.13 | |
| Probability of Firm Switch^5^ | 0.10 | 0.10 | 0.12 | 0.08 | 0.11 | 0.09 | 0.09 | 0.07 | 0.05 | 0.07 | 0.04 | 0.03 | |
| Probability of Industry Switch^6^ | 0.09 | 0.09 | 0.10 | 0.07 | 0.09 | 0.08 | 0.07 | 0.05 | 0.05 | 0.06 | 0.04 | 0.03 | |
| Probability of Occupation Switch^7^ | 0.19 | 0.31 | 0.30 | 0.15 | 0.21 | 0.19 | 0.15 | 0.15 | 0.22 | 0.11 | 0.12 | 0.13 | |
| All experiences are averages measured in years; ^1^Net annual income after tax in Euros; ^2^Proportion of sales managers that join from a different firm; ^3^Proportion of sales managers that join from a different industry; ^4^Proportion of sales managers that had managerial job just before joining; ^5^Probability of firm switch within the next year; ^6^Probability of industry switch within the next year; ^7^Probability of occupation switch within the next year; | | | | | | | | | | | | |  |
